# Multiobjective V_H_H discovery through integrated high-throughput screening and AlphaFold3-guided structural prioritization

**DOI:** 10.1126/sciadv.aef5325

**Published:** 2026-09-30

**Authors:** Max D. Overath, Suthimon Thumtecho, Esperanza Rivera-de-Torre, Melisa Benard-Valle, Darian S. Wolff, Rahmat Grahadi, Kasper H. Björnsson, Andreas S. H. Rygaard, Nils Hofmann, Jann Ledergerber, Anne Ljungars, Andreas H. Laustsen, Simon Olsson, Thomas J. Fryer, Timothy P. Jenkins

**Affiliations:** ^1^ Department of Biotechnology and Biomedicine, Technical University of Denmark, Kongens Lyngby, Denmark.; ^2^ Center for Translational Protein Design, Technical University of Denmark, Kongens Lyngby, Denmark.; ^3^ Division of Toxicology, Department of Medicine, Chulalongkorn University and King Chulalongkorn Memorial Hospital, Thai Red Cross Society, 10330 Pathumwan, Bangkok, Thailand.; ^4^ Novonesis, Kongens Lyngby, Denmark.; ^5^ Department of Biology, Faculty of Mathematics and Natural Sciences, Universitas Brawijaya, Malang, Indonesia.; ^6^ Molecular Design Laboratory, ETH Zurich, Zurich, Switzerland.; ^7^ Department of Computer Science and Engineering, Chalmers University of Technology and University of Gothenburg, Gothenburg, Sweden.; ^8^ IMES, Massachusetts Institute of Technology, Cambridge, MA, USA.

## Abstract

Finding therapeutic antibodies that bind multiple related targets with high affinity and favorable biophysical properties remains challenging and resource-intensive. For snakebite antivenoms, this challenge is critical as treatments must neutralize toxins across multiple snake species. We developed a pipeline combining high-throughput yeast screening, deep sequencing, and AlphaFold3 structure prediction to identify polyspecific variable domains of heavy chain–only antibodies (V_H_Hs) against long-chain α-neurotoxins. Multiplexed yeast display screening generated a dataset of diverse hits with varying binding specificities. AlphaFold3-generated V_H_H-toxin complex predictions enabled stringent high-precision structural triage of polyspecific V_H_Hs that bind conserved epitopes across multiple toxins, with a representative subset experimentally confirmed to block toxin binding to the acetylcholine receptor. These structural insights provided a starting point for computational optimization of affinity and soluble expression of the V_H_Hs, with experimental validation confirming that optimized V_H_H variants maintained broad binding specificity across toxins. This integrated approach supports structure-guided prioritization of polyspecific V_H_H hits, offering a high-precision filter that could reduce future reliance on extensive experimental specificity screening and providing a framework applicable to other therapeutic contexts, in which substantial antigen variation occurs and broad neutralization is essential.

## INTRODUCTION

Therapeutic antibodies have become well-established treatment modalities, widely used to manage and treat a variety of diseases, including cancers, autoimmune disorders, and infectious diseases, by specifically targeting and neutralizing disease-associated antigens (*1*). Advances in experimental workflows and computational methods, particularly machine learning, have streamlined antibody discovery and development in recent years (*2*–*5*). Despite this progress, antibody discovery and development remain a time- and resource-intensive endeavor. To deliver antibodies for therapeutic use, it is key to optimize for affinity and specificity against a desired epitope across a defined target homolog space while also mitigating off-target binding (*6*). These criteria often necessitate multiple rounds of selection to yield optimal leads, a time- and resource-intensive process that is especially limiting for complex target spaces in which binding across multiple protein homologs is required for therapeutic efficacy, as in the development of snakebite antivenom.

Snakebite envenomation is a devastating health burden, which causes an estimated 100,000 deaths annually (*7*), underscoring the urgent need for improved therapies against this disease. Recombinant antibodies have emerged as a promising alternative to traditional plasma-derived antivenoms (*8*–*11*), as it is hypothesized that recombinant antivenoms can be developed to become safer and more efficacious than their traditional counterparts (*12*, *13*). However, the discovery and development of these therapeutics come with several challenges. To develop polyspecific recombinant antivenoms with broad species coverage, a central objective is to identify broadly neutralizing antibodies that retain polyspecific binding to conserved neutralizing epitopes on toxin homologs across venoms from multiple snake species (*14*). Here, polyspecificity denotes breadth of specific binding across toxin variants, whereas broad neutralization denotes breadth of functional inhibition. Polyspecific binding to nonneutralizing epitopes may not confer protective activity. Beyond broad neutralization, optimal antibody candidates that are to be formulated into recombinant antivenoms should exhibit high solubility and thermal stability, which facilitate long shelf-life and might be beneficial for their deployment in remote areas with complex supply chains. Last, given that snakebite envenomation disproportionately affects low-income regions, cost-effective discovery and production methods are essential to ensure accessibility and affordability (*8*, *15*, *16*).

In this study, we sought to overcome key antibody discovery challenges such as obtaining polyspecificity, high affinity, and high solubility by developing a unified, high-throughput pipeline that seamlessly integrates antibody display technologies, deep sequencing, and in silico modeling for hit identification and optimization (Fig. 1). We applied this pipeline to the discovery of polyspecific variable domains of heavy chain–only antibodies (V_H_Hs) against long-chain three-finger α-neurotoxins (LNTxs). These highly lethal toxins are typically found in elapids, such as cobras and mambas (*17*, *18*), and bind and inhibit acetylcholine receptors (AChRs) (*19*), causing paralysis and muscle failure in victims and prey (*20*). They are therefore highly medically relevant targets for the discovery of broadly neutralizing antibodies (*10*, *11*, *21*, *22*) and other protein-based binders (*23*) that can be used to develop recombinant antivenoms. We deliberately chose a V_H_H library for this study, as this antibody format offers distinct advantages over full-length immunoglobulin G, such as higher stability, higher neutralization capacity per mass, and potential for low-cost manufacture, making them especially suitable for use in the context of snakebite envenomation (*12*, *14*, *24*, *25*).

**Fig. 1.**
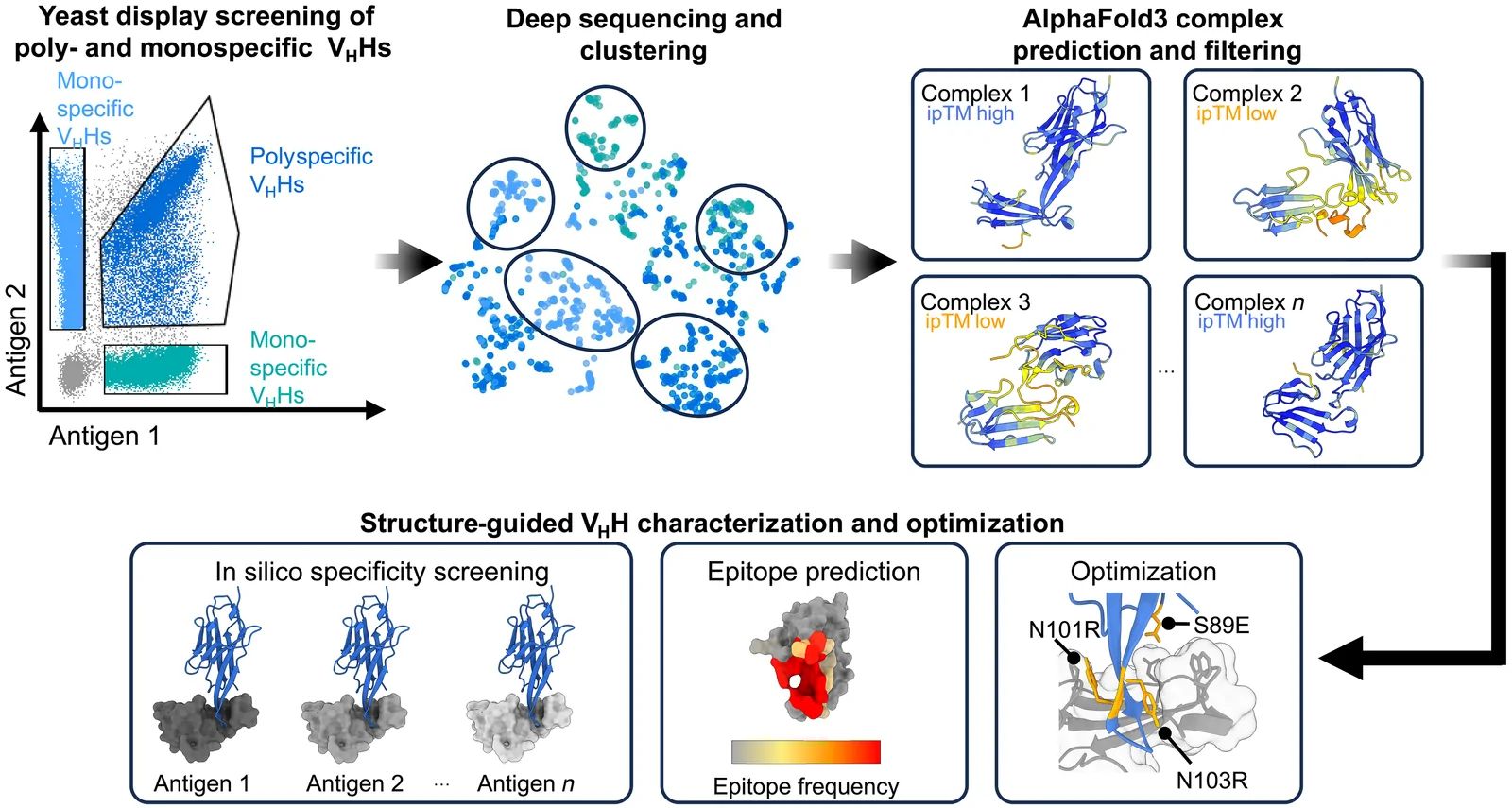
High-throughput and structure-guided pipeline for the discovery of polyspecific V_H_Hs. Multiplexed yeast display is used for the high-throughput screening of poly- and monospecific V_H_Hs against different LNTxs. The output clone pools are deep sequenced and clustered on the basis of CDR1-CDR3 sequence similarity to downsample and identify relevant V_H_H clones. AF3 is then used to predict complexes of V_H_Hs and their respective LNTxs with subsequent scoring of the complexes using the ipTM score to select high-confidence predictions. These structures support in silico specificity screening and enable structure-guided selection, characterization, and optimization of polyspecific V_H_Hs.

To enable the discovery of polyspecific V_H_Hs, we developed a streamlined dual-display approach that combined phage and yeast display, as this combination has previously been shown to effectively integrate the strengths of both techniques (*26*–*28*). Phage display enables efficient selection of binders from large libraries, while yeast display enables quantitative, multiplexed screening capabilities. We used multiplexed yeast display to efficiently identify polyspecific V_H_Hs binding to distinctly fluorescently labeled toxins. Following the selection and screening campaign, we integrated deep sequencing of all sorted populations (polyspecific, monospecific, and nonbinding) to build a comprehensive dataset of all antibody variants. This allowed for sequence clustering on the basis of complementarity-determining region (CDR) sequence similarity to reveal the diversity and relationships within the antibody library (*29*, *30*). When applied to both positive and negative selections, as done in this study, deep sequencing further enables improved identification of relevant antibody clones by normalization on the basis of the enrichment ratios (*31*). Collection of both positively and negatively labeled sequence data also greatly facilitates the downstream training, validation, and/or fine-tuning of antibody-specific machine learning models (*32*–*36*).

Recent advances in antibody-antigen structure prediction, particularly with AlphaFold3 (AF3), enable more accurate modeling of biomolecular complexes at scale (*37*). Here, we integrate AF3 systematically into a unified, high-throughput antibody discovery pipeline to support structure-guided hit triage, identification of epitope specificity, prioritization of hits targeting functional regions, and extensive in silico screening for polyspecificity across toxin homologs. We further use these antibody-antigen structures as starting points for computational optimization, improving affinity with a structure-informed language model (*38*) and solubility with CamSol (*39*), followed by experimental validation using a split-luciferase expression and binding assay. Together, this framework provides a scalable route to find and optimize V_H_Hs and other antibody therapeutics for snakebite envenomation and other indications.

## RESULTS

### Multiplexed yeast display and deep sequencing enable high-throughput discovery of poly- and monospecific V_H_Hs against LNTxs

We established an experimental pipeline for the high-throughput selection of poly- and monospecific V_H_Hs, integrating phage display biopanning and multiplexed yeast display. The starting point for this study was an enriched V_H_H library obtained from one round of phage display biopanning against a consensus LNTx (cLNTx), designed to capture conserved features shared across multiple long neurotoxins (*40*). The initial library was derived from camelid immune libraries generated by immunization with whole snake venoms, as described previously (*12*). Following a single round of phage display biopanning against the cLNTx, the enriched library was shuttled to a yeast display platform for high-throughput screening of poly- and monospecific V_H_Hs. On the basis of this yeast display library, we used three different fluorescence-activated cell sorting (FACS) strategies using three different toxins, namely the cLNTx, α-cobratoxin (αCbtx; UniProt ID: P01391), and α-bungarotoxin (αBgtx; UniProt ID: P60615) as antigens (Fig. 2A). Notably, αCbtx and αBgtx were both not considered for the design of the cLNTx. However, αCbtx is structurally closely related to the cLNTx [root mean square deviation (RMSD) = 1.25 Å], while αBgtx is more distant to both αCbtx (RMSD = 3.67 Å) and the cLNTx (RMSD = 3.01 Å) (Fig. 2A). At the primary sequence level, cLNTx shares 73.24 and 56.94% identity with αCbtx and αBgtx, respectively, while αCbtx and αBgtx share 54.93% between them. In a first yeast display screening round, we used FACS to simultaneously isolate cLNTx binding and cLNTx nonbinding V_H_H populations. Fluorescently labeled cLNTx was used to detect binding and SpyCatcher-GFP (green fluorescent protein) to detect V_H_H-SpyTag proteins displayed on yeast cells (Fig. 2B). This yielded enriched populations for cLNTx binding (cLNTx+ from 8.75 to 56.5%) and cLNTx nonbinding (cLNTx−/V_H_H display+ from 23.5 to 58%), respectively. Next, we used a polyspecific sort strategy by using two different fluorescent labels for αBgtx and αCbtx (Fig. 2C). Here, we could enrich three populations: αBgtx monospecific binding (αBgtx+/αCbtx− from 0.093 to 31.3%), αCbtx monospecific binding (αBgtx−/αCbtx+ from 7.74 to 49.5%), and αBgtx-αCbtx polyspecific binding (αBgtx+/αCbtx+ from 1.29 to 53.3%). In addition, we sorted the library for mono- and polyspecific V_H_Hs against cLNTx and αCbtx (fig. S1A). This resulted in enriched populations for cLNTx monospecific binders (cLNTx+/αCbtx− from 1.03 to 56.4%) and cLNTx-αCbtx polyspecific binders (cLNTx+/αCbtx+ from 6.44 to 34.5%). A population of αCbtx monospecific binders (cLNTx−/αCbtx+) could not be enriched.

**Fig. 2.**
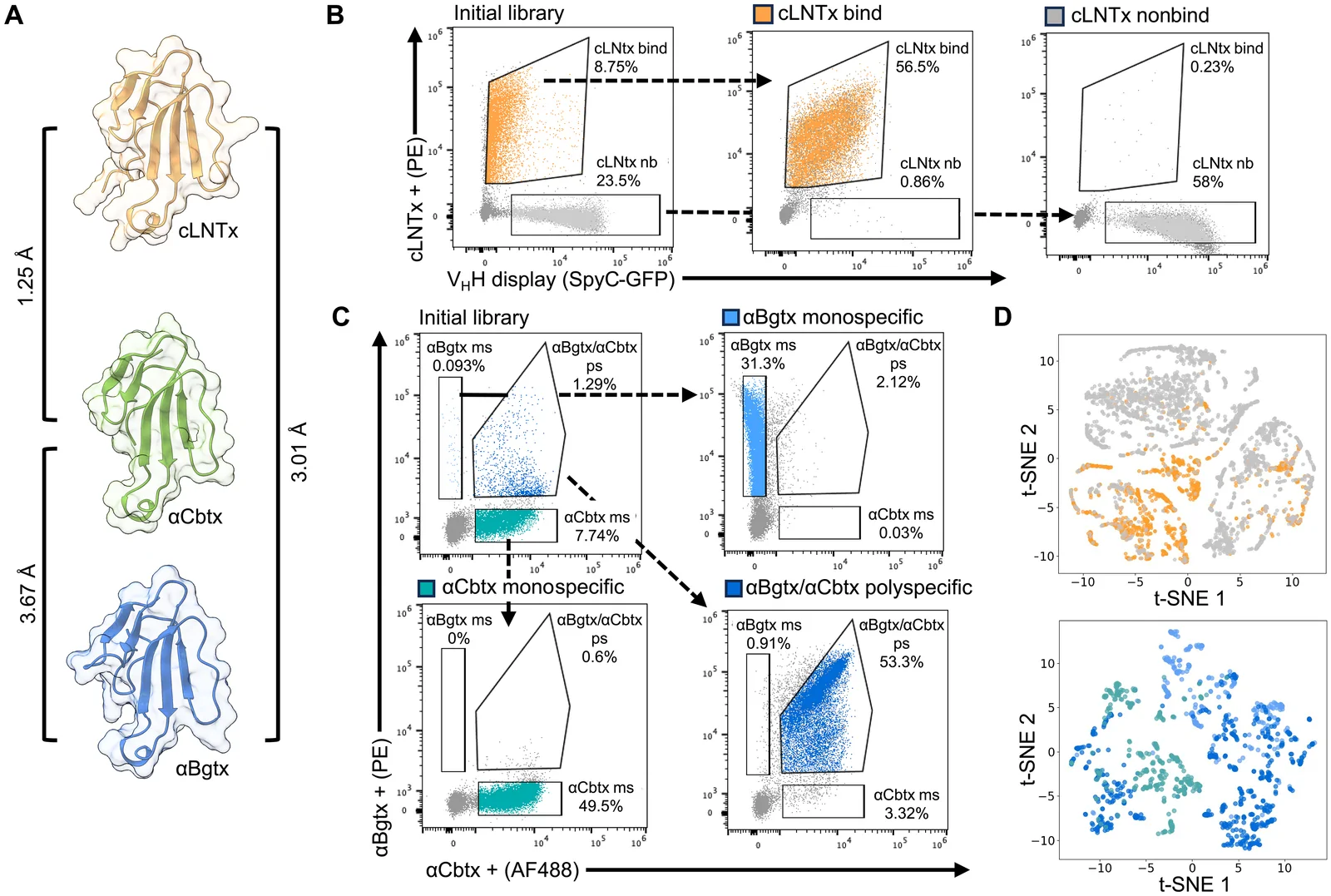
Yeast display guided screening of poly- and monospecific V_H_Hs. (**A**) Overview of LNTxs used for the screenings, including a cLNTx (structure predicted with AF3), αCbtx [PDB ID: 1YI5 (*44*)], and αBgtx [PDB ID: 1HC9 (*55*)] with RMSD between structures annotated. (**B**) Yeast display screenings were performed using biotinylated cLNTx with streptavidin-PE to detect antigen binding and SpyCatcher-GFP to detect V_H_H surface display. Yeast cells were sorted with FACS into cLNTx binder and nonbinder populations on the basis of these two signals. (**C**) Poly- and monospecific selections were performed using biotinylated αBgtx detected with streptavidin-PE and αCbtx directly labeled with AF488. On the basis of these two signals, three populations were FACS sorted: αBgtx monospecific V_H_Hs, αCbtx monospecific V_H_Hs, and αBgtx/αCbtx polyspecific V_H_Hs. (**D**) Each yeast display selection was deep sequenced, and sequences were clustered by hierarchical clustering using pairwise distances across CDR1-CDR3. Clusters were visualized using *t*-distributed stochastic neighbor embedding (*t*-SNE) plots, enabling selection of relevant variants for each sample. Coloring of populations is the same as in (B) and (C).

We then deep sequenced the enriched yeast display populations. Sequencing reads from paired sorted outputs for each selection (cLNTx binder versus nonbinder, cLNTx/αCbtx poly- versus monospecific, and αBgtx/αCbtx poly- versus monospecific) were processed using an enrichment-based filter to prioritize sequences supported by the selection. The resulting V_H_H sequences were clustered by hierarchical clustering using pairwise distances computed from global alignments of concatenated CDR1-CDR3 sequences, enabling identification of diverse hit families for downstream AF3 complex prediction (Fig. 2D and fig. S1B). Most clusters were dominated by sequences from a single-sorted output (fig. S2), suggesting that the selections enriched distinct CDR1-CDR3 sequence families that may correspond to different binding behaviors. To ensure the broad coverage of sequence space, we selected at least two V_H_Hs per cluster by random sampling, ensuring that each cluster contributed hits for AF3 complex predictions (fig. S3).

### AF3 enables high-confidence prediction of multiple V_H_H-toxin complexes

Classically, the next step in antibody discovery is to screen hits for affinity and specificity and to map their epitopes to identify the binding site and assess functional relevance. In practice, epitope mapping is typically performed either by solving antibody-antigen complex structures or by mutational scanning approaches such as alanine scanning; both can be resource-intensive and slow and often become a bottleneck when many hits must be evaluated. Subsequently, top hits are engineered to optimize properties such as affinity, polyspecificity, solubility, and other developability-related biophysical parameters. Accurate antibody-antigen structure prediction would greatly streamline these stages. Recent structure prediction models, in particular AF3, have improved on predicting antibody-antigen complexes, but performance remains imperfect (*37*), and it has not yet been demonstrated how reliably such predictions can be integrated into an end-to-end antibody discovery and engineering pipeline. To investigate whether AF3 had the ability to overcome these shortcomings and accurately predict high-confidence V_H_H-LNTx complexes, we evaluated AF3 predictive performance on a structure of a V_H_H [TPL1158_01_C09 (C09)] in complex with αCbtx, which we had previously described (Fig. 3A). C09 originated from the same phage display library used for this study, and the complex structure of C09 with αCbtx was resolved by x-ray crystallography [Protein Data Bank (PDB) ID: 9GCN] (*40*). It is worth mentioning that this structure was released after the AF3 training data cutoff and was not used as a template for predictions. As such, it should present an unbiased example of AF3’s ability to accurately predict V_H_H-LNTx complexes. Encouragingly, we found that the predicted complex aligned closely with the crystal structure (RMSD = 0.902 Å; DockQ = 0.813) and showed an identical binding mode of the CDR3 loop (Fig. 3A). Given that C09 was also shown to be polyspecific against four additional LNTxs, AF3 capabilities to predict this polyspecificity were also benchmarked, as well as differentiating between binding and nonbinding interactions using six nontarget three-finger toxin decoys (three short-chain α-neurotoxins and three cytotoxins; Fig. 3B). To score predicted complexes, we used the interface predicted template modeling (ipTM) score. We used ipTM >0.8 as a conservative high-confidence threshold, consistent with published guidance on interpreting ipTM for multimer/complex predictions (*41*). This choice is also consistent with prior antibody design studies reporting that higher AF3 ipTM values are enriched among experimentally successful binders and that applying ipTM-based filters can improve hit rates (*42*, *43*). Consistently, every C09:LNTx complex achieved a score above the threshold where expected, whereas all decoy targets exhibited ipTM values well below this threshold, strongly suggesting that AF3 can discriminate between binders and nonbinders.

**Fig. 3.**
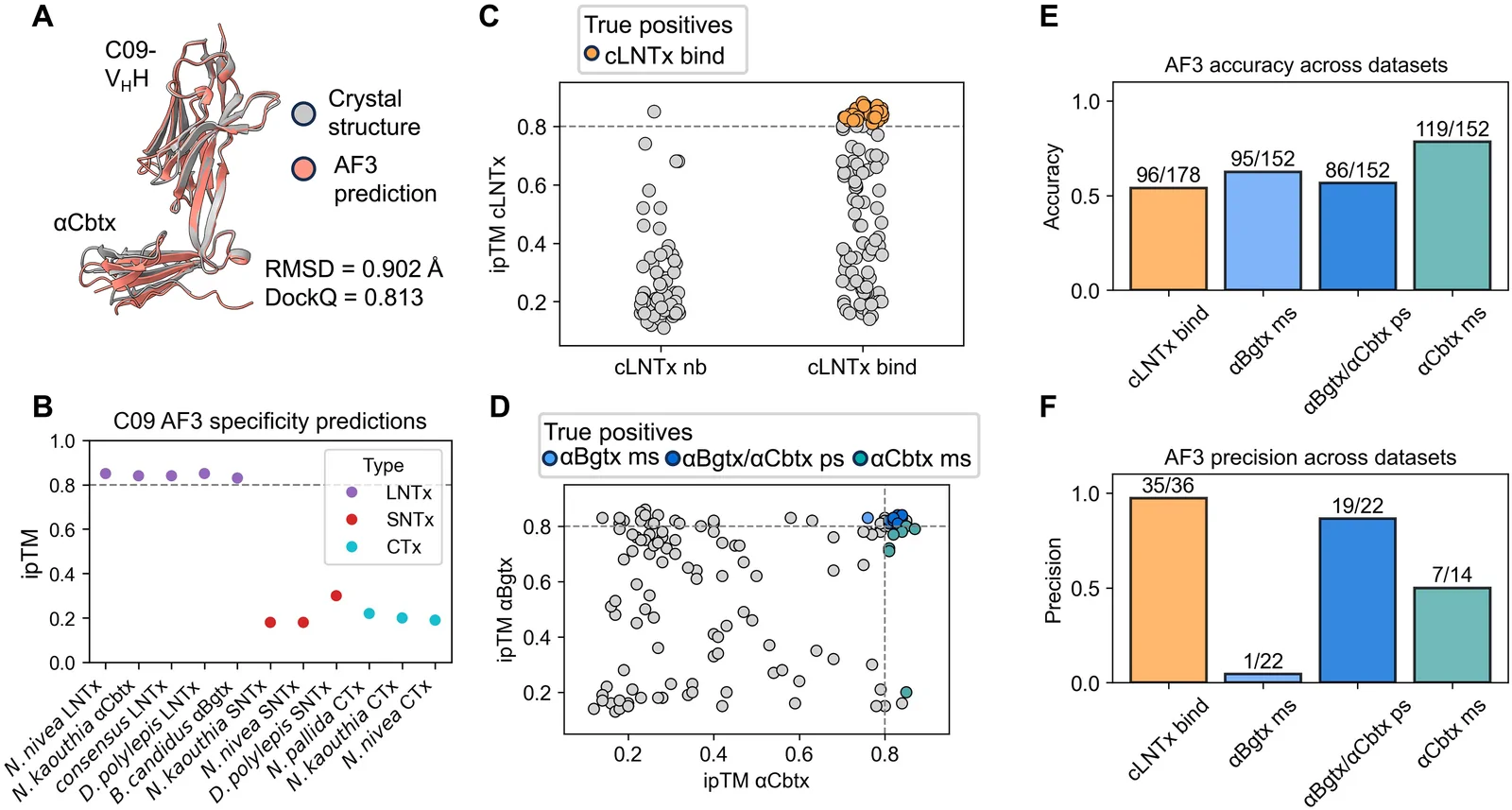
AF3 predictions of V_H_Hs in complex with LNTxs. (**A**) The accuracy of AF3 prediction is benchmarked using a previously resolved crystal structure of a V_H_H [TPL1158_01_C09 (C09)] in complex with αCbtx [PDB ID: 9GCN (*40*)], demonstrating high alignment between predicted and resolved structures (RMSD = 0.902 Å; DockQ = 0.813). (**B**) AF3 enables the prediction of experimentally validated polyspecificity of C09 with four other LNTxs as well as nonbinding to nontargets used as decoys [three short-chain α-neurotoxins (SNTx) and three cytotoxins (CTx)] using the ipTM as a scoring parameter (threshold: 0.8). (**C**) AF3 was used to cofold 178 V_H_H sequences from the cLNTx binder and nonbinder populations together with the cLNTx sequence. Structures with an ipTM confidence score greater than 0.8 were labeled as predicted binders, and true positives are color coded. (**D**) From the αBgtx/αCbtx poly/monospecific dataset, 152 V_H_H sequences were selected and cofolded with αBgtx and αCbtx sequences in separate AF3 predictions. Predictions were labeled as correct when the predicted binding pattern based for both toxins matched the yeast display selection category for each V_H_H. (**E** and **F**) Accuracy and precision of predicted mono- and polyspecificity and true experimental binding based on the sequencing data (nb, nonbind; ps, polyspecific; ms, monospecific).

To further evaluate whether AF3 could accurately predict V_H_H-LNTx complexes and therefore streamline hit prioritization, we used AF3 to model structures for V_H_H sequences selected from our clustered deep-sequencing data. As before, we used an ipTM score >0.8 as our binding threshold and benchmarked AF3’s predictions against experimental binding data from yeast display screening across three datasets. AF3 demonstrated strong precision in identifying true binders. While AF3 high-confidence predictions (ipTM >0.8) showed strong precision, many experimentally validated binders fell below this threshold, resulting in moderate overall accuracy (Fig. 3, C to F). To evaluate the reproducibility of AF3 predictions, a subset of V_H_H-toxin pairs was modeled using five different random seeds. Predictions with ipTM scores above 0.8 showed negligible variation between runs, indicating that high-confidence predictions were highly stable and reproducible (fig. S4). Notably, AF3 performance varied by binding specificity. For the cLNTx dataset (178 V_H_H sequences), AF3 identified 36 predicted binders, of which 35 were present in the sorted cLNTx binding population (precision = 0.97). For the αBgtx/αCbtx dataset (152 sequences), AF3 excelled at identifying polyspecific binders (19 of 22 correct; precision = 0.86) but struggled with monospecific binders, particularly for αBgtx (1 of 22 correct; precision = 0.04). The cLNTx/αCbtx dataset further confirmed this trend, with perfect precision for polyspecific binders (23 of 23 = 1.00) but lower precision for monospecific hits (9 of 15 = 0.60) (fig. S5). AF3 flagged 74 unique V_H_H sequences as high-confidence binders across all screening datasets (258 evaluated). Overall, polyspecific predictions achieved a precision of 0.93 (42 of 45) compared with 0.33 (17 of 51) for monospecific predictions. These results demonstrate that AF3 can be effectively integrated into the discovery workflow to generate high-confidence complex structures and prioritize polyspecific V_H_Hs. However, recall remained low across all groups (polyspecific: 0.24, 42 of 176; monospecific: 0.18, 17 of 97), and the challenges in mono/polyspecific discrimination indicate that AF3 is best used as a stringent filter for confident binders rather than a comprehensive screening tool.

### AF3 predictions enable epitope characterization and are experimentally confirmed

Given that there was a strong correlation between high-confidence predictions of AF3 and experimental binding data, we reasoned that the predicted high-confidence complexes were accurate enough to analyze epitopes and binding modes across the different selections. One hypothesis we focused on was that toxin epitopes targeted by monospecific V_H_Hs would likely be distinct from V_H_Hs that are polyspecific against different LNTxs. We thus aligned the AF3-obtained true-positive complexes for each V_H_H selection on the basis of the respective LNTx structure (Fig. 4A). The cLNTx binding complexes revealed two distinct clusters of non-overlapping epitopes and three major V_H_H binding modes. The αBgtx/αCbtx polyspecific selection consists of a single non-overlapping epitope shared by all V_H_Hs. Similarly, the αBgtx monospecific and αCbtx monospecific V_H_Hs bound to the same non-overlapping epitope, although the binding mode of αCbtx monospecific V_H_Hs was distinct from that of αBgtx/αCbtx polyspecific V_H_Hs. These findings substantiated our initial hypothesis of distinct mono- versus polyspecific epitopes.

**Fig. 4.**
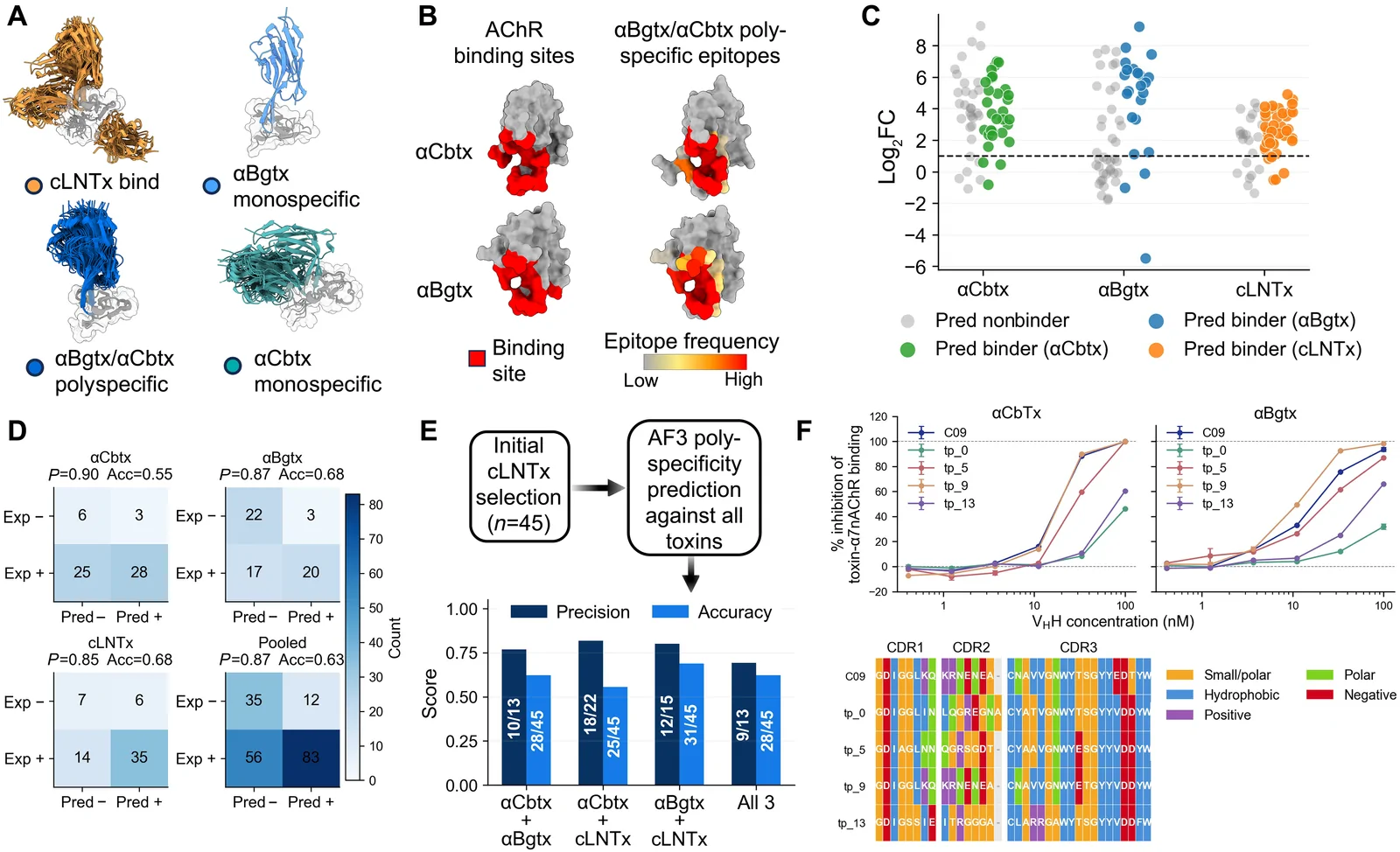
Characterization and experimental validation of AF3-predicted complexes. (**A**) Representative AF3-predicted V_H_H-toxin complexes for true-positive predictions (ipTM >0.8) from each yeast display sample: cLNTx binding, αBgtx monospecific, αBgtx/αCbtx polyspecific, and αCbtx monospecific. Toxins are shown in gray. cLNTx binding samples reveal three major V_H_H binding modes that converge on two epitope clusters. The αBgtx monospecific V_H_H engages an overlapping epitope and adopts a similar binding mode to the αBgtx-αCbtx polyspecific V_H_Hs. αCbtx monospecific V_H_Hs target a similar epitope region but with a distinct binding mode. (**B**) Comparison of polyspecific V_H_H epitopes with AChR-binding sites of LNTxs. Binding sites of αCbtx and αBgtx with the AChBP [PDB ID: 1YI5 (*44*)] are colored red. Epitope residues from true-positive αBgtx/αCbtx polyspecific V_H_Hs are colored by epitope frequency. (**C**) Split-luciferase binding signals (log_2_FC over the background) for 62 tested V_H_Hs against cLNTx, αCbtx, and αBgtx. Experimental binding was defined as log_2_FC > 1. Points are colored by AF3-predicted binders (ipTM >0.8) for the corresponding toxin; gray indicates predicted nonbinders. The dashed line marks the binding threshold. (**D**) Confusion matrices comparing AF3 predictions with experimental split-luciferase binding for each toxin and pooled across toxins. Cells show V_H_H-toxin pair counts; precision (P) and accuracy (Acc) are reported above each matrix. (**E**) Retrospective evaluation of AF3 as a filter for polyspecific binders using the cLNTx-selected binders and nonbinders (*n* = 45). Performance is summarized for predicted binding to toxin pairs or all three toxins. (**F**) Dose-response inhibition of toxin α7nAChR binding by five αBgtx/αCbtx polyspecific V_H_Hs against αCbtx and αBgtx (*n* = 3). CDR sequences of the tested V_H_Hs are shown colored by physicochemical properties. V_H_Hs were selected to represent CDR3 sequence diversity on the basis of AF3 complex predictions. C09 is included as a broadly neutralizing V_H_H control.

The alignment of in vitro and in silico data thus gave us sufficient confidence to further investigate the predicted structural interactions and actively evaluate whether a given V_H_H would not just bind a target toxin but would likely also functionally inhibit it. Given LNTxs’ native mode of action involving binding to and inhibiting the nicotinic AChR (nAChR) we could directly assess whether the V_H_H-toxin epitopes overlapped with the nAChR binding sites. In practice, we used a crystal structure of αCbtx bound to acetylcholine binding protein (AChBP) (*44*) as a reference and overlaid the polyspecific αCbtx-V_H_H and αBgtx-V_H_H complexes to identify interaction residues (defined as those within 4 Å of the AChBP structure; Fig. 4B, left panel). The frequency of each toxin residue being targeted across all polyspecific V_H_Hs was calculated and mapped as a color gradient onto the LNTx structures (Fig. 4B, right panel). This comparison revealed that the epitopes of αCbtx and αBgtx binding V_H_Hs overlap with the receptor binding site, i.e., they would have high likelihood to functionally inhibit toxic activity by outcompeting the toxin for receptor binding. We also quantified epitope conservation by direct sequence comparison, revealing that polyspecific V_H_Hs preferentially target conserved residues (67% identical or conservative substitutions versus 52% for monospecific V_H_Hs) while avoiding radical changes (18% versus 34%), suggesting an association between epitope sequence conservation and polyspecificity (fig. S6).

To validate that the obtained high-confidence structures reflect the predicted binding behavior, we selected a panel of V_H_Hs and experimentally tested them using DNA oligo pool assembly and split-luciferase assays. The panel was designed to probe AF3 performance across outcome classes and represent true positives (AF3-predicted binders consistent with yeast display selection), false positives (AF3-predicted binding but not enrichment by yeast display), false negatives (no AF3-predicted binding despite yeast display enrichment), and true negatives (no AF3-predicted binding and originating from the cLNTx nonbinding selection). In total, 62 unique V_H_Hs were tested for binding against cLNTx, αCbtx, and αBgtx (Fig. 4C). We defined binding as split-luciferase log_2_ fold change (log_2_FC) >1 (see Materials and Methods and fig. S7) and AF3 ipTM >0.8, then evaluated predictive performance using confusion matrices per toxin, and pooled across all V_H_H-toxin pairs (Fig. 4D). Consistent with the trends observed from the yeast display selection, AF3 predictions showed high precision across toxins (αCbtx: 0.90; αBgtx: 0.87; cLNTx: 0.85; pooled: 0.87), whereas accuracy was lower (0.55, 0.68, and 0.68; pooled: 0.63). These results indicate that AF3-predicted binders were usually correct. However, this high precision comes with a trade-off in sensitivity: A substantial fraction of experimentally positive interactions was not recovered by the ipTM >0.8 cutoff (pooled false negatives = 56), highlighting that AF3 can serve as a stringent filter for confident binders but may miss many true experimental binders under a high-confidence threshold. Sensitivity analysis across ipTM thresholds confirmed that the 0.8 cutoff used throughout this study represents a reasonable but conservative operating point; lower thresholds such as 0.75 yield similar precision with improved recall (fig. S8).

To address the practical question of whether AF3 could have been used up front to enrich for polyspecific binders without additional yeast display screening, we performed a retrospective polyspecificity analysis restricted to the initial cLNTx binder/nonbinder selection (*n* = 45) as a proxy for the initial library after one round of phage display biopanning. Within this dataset, we evaluated AF3’s ability to identify V_H_Hs predicted to bind two or three of the toxins (ipTM >0.8) and benchmarked these predictions against experimental split-luciferase results (log_2_FC > 1), reporting precision and accuracy for each polyspecificity category (Fig. 4E). AF3 achieved high precision across categories, i.e., αCbtx and αBgtx, 10 of 13 (0.77); αCbtx and cLNTx, 18 of 22 (0.82); αBgtx and cLNTx, 12 of 15 (0.80); and all three toxins, 9 of 13 (0.69). Accuracy was again consistently lower, ranging from 0.56 to 0.69 across categories. This suggests that even without additional screening, AF3 would have provided a high-precision filter for prioritizing polyspecific V_H_Hs from the initial selection. Assay agreement analysis revealed strong concordance between yeast display and split-luciferase for polyspecific V_H_Hs but weak concordance for monospecific calls, particularly αBgtx and cLNTx monospecific binders (fig. S9). This label noise explains why AF3 performance appears lower for monospecific predictions, as many apparent failures reflect imperfect experimental labeling rather than AF3 limitations. We therefore emphasize polyspecific performance, benchmarked against split-luciferase validation, as the primary finding.

Last, to validate that the polyspecific V_H_Hs target the predicted receptor-overlapping epitope, we selected five V_H_Hs spanning the CDR3 diversity of AF3 high-confidence αBgtx/αCbtx polyspecific predictions and assessed their ability to inhibit toxin binding to a human α7 nicotinic AChR (α7nAChR) chimera (see Materials and Methods). This panel included C09, a V_H_H previously shown to be broadly neutralizing and used as a structural benchmark earlier in this study, serving as a positive control. All five V_H_Hs showed dose-dependent inhibition of both αCbtx and αBgtx binding to α7nAChR (Fig. 4F), substantiating the predicted receptor-overlapping epitopes. Overall, these results reaffirm how AF3 can be incorporated into the discovery process and demonstrate that ipTM values correlate with experimental binding and polyspecificity outcomes.

### Structure-guided optimization of V_H_Hs

While AF3 enabled us to identify likely binding epitopes and select V_H_Hs with high functional potential, the true power lies in its ability to provide a starting point for structural guided refinement of our V_H_Hs with epitope-paratope information. To this end, we used two previously described methods for antibody optimization on the 19 high-confidence complexes of αBgtx/αCbtx polyspecific V_H_Hs, given that they represented the most relevant V_H_Hs for the potential development of a broadly neutralizing antivenom. For solubility optimization, we applied the CamSol method (*39*), which proposes mutations predicted to enhance solubility. However, to avoid compromising binding, residues forming the paratope were excluded from mutation. For affinity optimization, we used a recently developed structure-informed language model (*38*) that suggests point mutations with potential to improve binding affinity. We therefore designed variants for each V_H_H against both αCbtx and αBgtx separately, retaining only mutations that were predicted to improve binding to both targets, thereby preserving polyspecificity.

Both approaches yielded mutations distributed across CDRs and framework regions (FWRs) (Fig. 5, A and B). Notably, mutations for improved affinity were enriched in FWR1 and FWR3, while CamSol mutations were predominantly located in CDR3 (fig. S10). In total, 37 CamSol-derived and 57 structure-evolution designs were screened using the split-luciferase assay described previously. Several structural evolution (se) variants exhibited markedly enhanced binding signals (up to fourfold improvement in split-luciferase signal) relative to the wild type (WT) for both αCbtx and αBgtx (Fig. 5C). Likewise, multiple CamSol variants demonstrated improved expression signals in the HiBiT assay, serving as a proxy for soluble protein expression (Fig. 5D). Notably, most CamSol variants, while improving expression, did not suffer from substantially reduced binding to αCbtx or αBgtx, potentially due to the structure-informed mutation scheme (fig. S11).

**Fig. 5.**
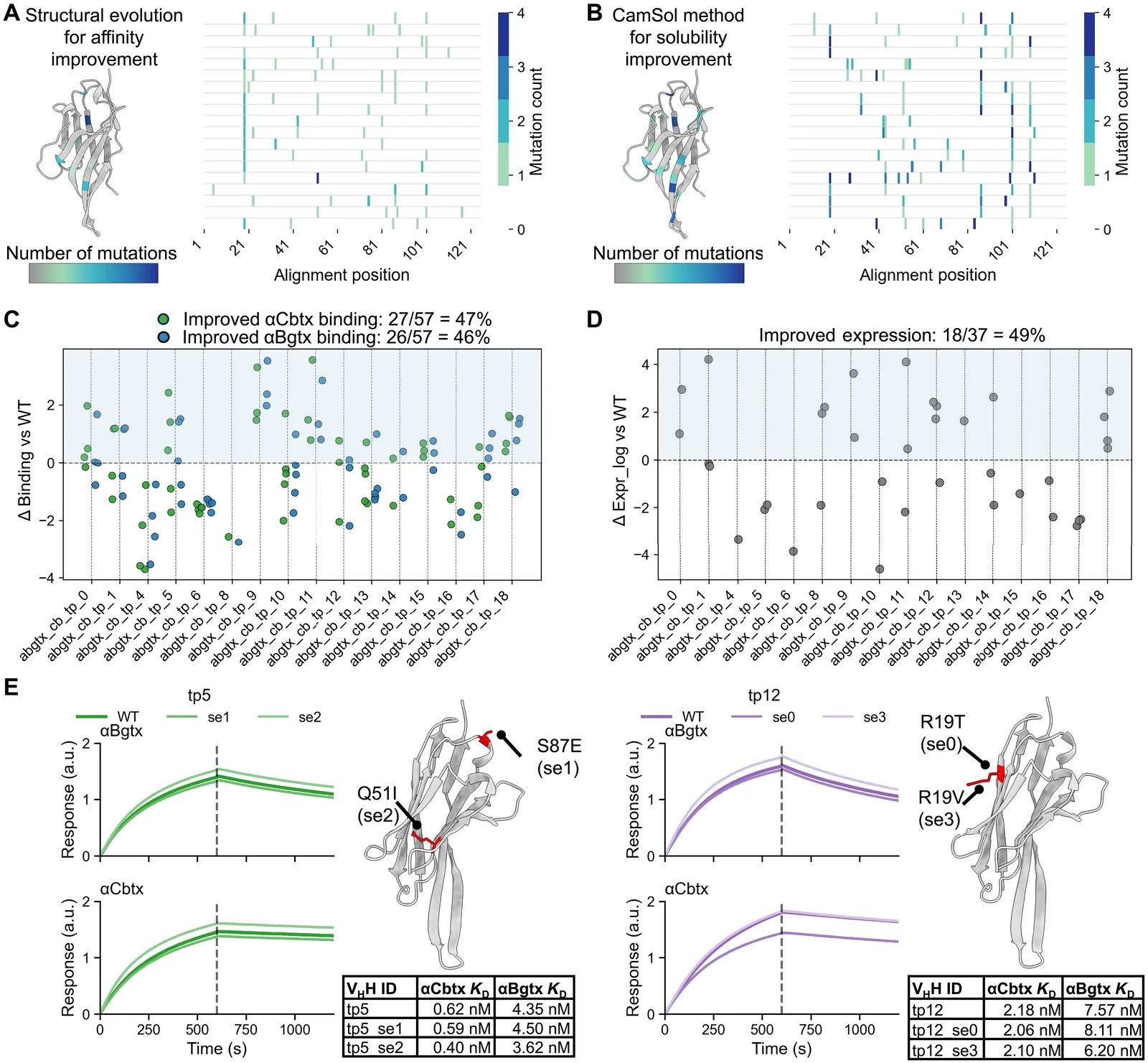
Structure-guided affinity and soluble expression optimization. (**A**) Mutational landscape of affinity-optimized variants generated from an initial set of 19 WT αBgtx/αCbtx polyspecific V_H_Hs. Each WT V_H_H was diversified using a structural evolution (se) model, and the resulting substitutions are summarized per alignment position (heatmap; color indicates mutation count) and mapped onto a reference V_H_H structure to highlight regions preferentially targeted during affinity optimization. (**B**) Mutational landscape of solubility-optimized variants generated from the same initial set of 19 WT αBgtx/αCbtx polyspecific V_H_Hs. Each WT V_H_H was diversified using CamSol for solubility enhancement, and substitutions are shown per alignment position and projected onto the same reference V_H_H structure to compare the distribution of mutations introduced by solubility-driven optimization. (**C**) Experimental validation of these variants using the split-luciferase assay, with binding measured against both αBgtx and αCbtx. Shown is the Δ binding of variants versus the WT binding for both αCbtx (green dots) and αBgtx (blue dots). (**D**) Experimental validation of the CamSol variants using the split-luciferase HiBiT assay for expression measurements. Shown is the Δ expression of variants versus the WT. (**E**) BLI binding kinetics for tp5 and tp12 WT and two se variants each. Association/dissociation sensorgrams are shown alongside fitted *K*_D_ (dissociation constant) values. Structures show the respective V_H_Hs, with mutated residues highlighted in red and each mutation annotated. a.u., arbitrary units.

To assess affinity improvements beyond the split-luciferase assay signal, we selected a panel of designed V_H_Hs together with their unmutated WT counterparts and measured their affinities for αCbtx and αBgtx using biolayer interferometry (BLI). We first demonstrated that the selected WT V_H_Hs already exhibited very high binding affinities for both targets ranging from 7.57 to 0.62 nM (fig. S12). BLI measurements indicated that most variants had affinities similar to, or modestly improved over, the WT. Several interactions were in the subnanomolar range, where kinetic parameter estimation can approach practical sensitivity limits and should therefore be interpreted cautiously. For example, tp5_se2 showed an apparent improvement against αCbtx (0.62 to 0.40 nM) and a modest shift against αBgtx (4.35 to 3.62 nM). Likewise, tp12_se3 exhibited an apparent modest improvement against αBgtx (7.57 to 6.20 nM) while maintaining comparable affinity to αCbtx (2.10 nM versus 2.18 nM) (Fig. 5E and fig. S13). Notably, all variants showing apparent improvements carried mutations in FWRs distant from the paratope, suggesting that the enhanced affinity may have arisen from improved folding and/or the stability of the V_H_H (Fig. 5E). While apparent affinity improvements were modest, these results demonstrate how structural information on antibody-antigen complexes obtained with AF3 can guide targeted antibody optimization.

## DISCUSSION

Multiobjective antibody discovery pipelines are becoming essential as therapeutic antibody development moves toward broader, faster, and more cost-efficient discovery cycles. Traditional workflows still rely on sequential screening and optimization, often requiring months to years of iteration to balance affinity, solubility, specificity, and other developability constraints. Here, we show that these objectives can be addressed in parallel by embedding structure prediction and in silico optimization directly into the early discovery phase. We established an integrated pipeline combining multiplexed yeast display, deep sequencing, and AF3-based complex modeling to support structure-guided prioritization and optimization of polyspecific V_H_Hs against LNTxs.

Differentially fluorescently labeled toxins enabled simultaneous selection of mono- and polyspecific V_H_Hs, while deep sequencing provided comprehensive coverage of binder diversity and a benchmark dataset for model evaluation. This combination allowed the identification of polyspecific V_H_Hs at scale and created a foundation for integrating machine learning models into antibody selection workflows. A key finding of the pipeline was that AF3 can be used as a structural filter within the discovery process rather than purely as a post hoc characterization tool. In our hands, ipTM >0.8 yielded a high-precision set of experimentally confirmed V_H_H binders, consistent with its use as a confidence filter in prior antibody design workflows and independently validated across large-scale nanobody-antigen benchmarks (*42*, *43*, *45*). This high precision came at the cost of sensitivity, with a substantial fraction of experimentally validated V_H_H binders falling below the threshold. Retrospective analysis suggested that lower thresholds, such as 0.75, recover more true binders with modest precision loss, although broad conclusions are difficult to make given the limited size of the dataset, and the 0.8 threshold remains well supported in the literature. AF3 therefore distinguished functional binders from nonbinders with high precision, particularly for polyspecific V_H_Hs. Our results also suggested that polyspecific hits can be prioritized directly after an initial enrichment step by using AF3 predictions across multiple toxins. Although this analysis was limited to a subset (*n* = 45), it indicates a potential route to reducing future screening efforts. A subset of polyspecific V_H_Hs was confirmed to inhibit αCbtx and αBgtx binding to nAChR in a dose-dependent manner, substantiating that they target functional toxin-binding sites and positioning them as potential leads for recombinant antivenom development. Predictive performance was lower for monospecific V_H_Hs, reflecting both current limitations of AF3 in modeling antibody-antigen complexes and, in the case of αBgtx and cLNTx monospecific V_H_H binders, imperfect initial screening during yeast display. Moreover, the stringent ipTM threshold prioritizes precision over sensitivity, and a substantial fraction of experimentally validated V_H_H binders fell below ipTM >0.8. Increasing the number of sampled complex predictions and adopting ensemble-based approaches have been shown to improve multimer prediction (*46*, *47*) and are likely to further enhance performance in this setting.

Having access to structural information early in the discovery campaign enables a more rational antibody engineering approach. We used AF3-generated complexes as starting points to guide targeted optimization of affinity and solubility with a structure-informed language model (*38*) and the CamSol algorithm (*39*). Parallel screening of the V_H_H variants in a split-luciferase assay rapidly identified V_H_H designs with improved binding signals or expression used as proxy for solubility. Notably, many beneficial substitutions for the structure-informed language model fell outside the CDRs, consistent with the structure-informed language model’s premise that conditioning on the full antibody-antigen complex can prioritize mutations that preserve or enhance the overall complex stability, including framework changes distal from the antibody-antigen interface. While split-luciferase screening identifies relative binding improvements, it does not provide quantitative affinity measurements. The V_H_Hs selected for optimization already exhibited subnanomolar to low-nanomolar affinities, underscoring the strength of the upstream discovery workflow while simultaneously limiting the scope for further affinity improvements and making additional optimization intrinsically more challenging. In this context, the optimization stage should be viewed primarily as a proof of principle that predictive models can be coupled to high-throughput experimental readouts within the same pipeline to tackle multiple objectives in parallel.

Several challenges remain. Some experimentally validated V_H_H binders did not yield high-confidence AF3 predictions, highlighting current limitations of AF3 for accurate antibody-antigen complex prediction that may be at least partially mitigated by more extensive conformational sampling and/or explicit modeling of alternative binding modes. Furthermore, high ipTM confidence does not always correlate with correct binding pose (*48*), underscoring that AF3 is best used as a triage filter rather than a guarantee of structural accuracy. While the V_H_H format in combination with small, structurally well-defined toxins is an attractive test case, full-length antibodies and larger, more flexible targets remain difficult for current structure prediction models.

In summary, this study illustrates how coupling structure prediction with high-throughput selection and screening can help move antibody discovery from a sequential process to a more parallelized and predictive process. By integrating multiplexed yeast display, deep sequencing, AF3 complex modeling, and structure-guided optimization into a single workflow, we provide a scalable foundation for structure-guided prioritization and optimization of V_H_Hs and other antibody formats. Looking ahead, recent repertoire-scale structural profiling of tens of thousands of V_H_Hs using a similar AF3-based framework (*45*) supports the approach presented here and demonstrates a path toward full library-scale triage. Emerging open-source models, such as Protenix-v2 (*49*), reporting improved performance over AF3, may further facilitate broader adoption of these types of pipelines. We anticipate that similar approaches will be useful not only for recombinant antivenom development but also for therapeutic, diagnostic, and biotechnological applications where rapid discovery of antibodies with tailored functional and developability profiles is required.

## MATERIALS AND METHODS

### Toxins

The long-chain three-finger α-neurotoxin (cLNTx) was produced in *Komagataella phaffii* KM71H as previously described (*40*). Lyophilized forms of αCbtx (L8114) and αBgtx (L8115) were obtained from Latoxan SAS (Portes-lès-Valence, France) and resuspended in phosphate-buffered saline (PBS) and quantified by absorbance at 280 nm (*A*_280_). Biotinylation of toxins was done using the Innolink Biotin 354S kit (Sigma-Aldrich, Germany), and fluorescence labeling of αCbtx was done using the Alexa Fluor 488 (AF488) NHS Ester Kit (Invitrogen A20000) following the manufacturer’s instructions.

### Cloning of phage display outputs into a yeast display vector

V_H_H genes in phagemids from previously described phage display biopanning against cLNTx (*40*) were amplified by polymerase chain reaction (PCR) (Q5 High-Fidelity 2X Master Mix, M0492S, New England Biolabs) introducing 30–base pair overhangs for in vivo assembly in yeast by homologous recombination. Primers were as follows: Homology_fwd: 5′-GGCGGGTCTGGTGGGGGAGGATCTGCCATGCAGGTGCAGCTGCAGGAG-3′; Homology_rev: 5′-TCGCTCGTACCTGGGGTTTCGCTAGCGGCCGCTGAGGAG-3′.

PCR products were purified using the GeneJET PCR Purification Kit (K0702, Thermo Fisher Scientific) following the manufacturer’s instructions. An in-house vector for yeast display containing a SpyTag, N-Terminal Aga2 (galactose-inducible) was digested using Nco I-HF (R3193L, New England Biolabs) and Not I-HF (R3189L, New England Biolabs), gel excised, and purified using the GeneJET Gel Extraction Kit (K0692, Thermo Fisher Scientific). PCR products and digested vectors were transformed into *Saccharomyces cerevisiae* EBY-100 strain by chemical transformation, as previously described (*50*).

### Production and purification of SpyCatcher-GFP

A SpyCatcher (*51*)-GFP fusion protein was recombinantly expressed in *Escherichia coli* BL21-(DE3) cells. The SpyCatcher-GFP sequence was encoded on the pET-SpyC-GFP plasmid including a N-terminal His_6_ tag and introduced into cells via chemical transformation. Expression was initiated by diluting 1 ml of a saturated overnight culture into 125 ml of 2xYT medium, followed by incubation at 37°C with shaking at 250 rpm for 2 hours. Induction was achieved by adding isopropyl-β-d-thiogalactopyranoside (IPTG) to a final concentration of 1 mM, and the culture was grown overnight at 25°C with shaking at 250 rpm. The cells were harvested the next day by centrifugation at 4800*g* for 20 min, and the resulting pellet was resuspended in 12.5 ml of equilibration/wash buffer (20 mM sodium phosphate, 500 mM NaCl, and 30 mM imidazole, pH 7.4). Cell lysis was performed using sonication, and the lysate was clarified by centrifugation at 4800*g* for 20 min. The soluble fraction was used for purification using immobilized metal affinity chromatography with a Ni-NTA resin. Following sample loading, the column was washed with an equilibration/wash buffer to remove nonspecifically bound proteins. The target protein was eluted using equilibration/wash buffer + 500 mM imidazole.

### Yeast display selections

Transformed yeast cells were cultured in a selective YNB (yeast nitrogen base)-CAA (casamino acids) medium containing 2% (w/v) glucose at 220 rpm and 30°C for 12 to 16 hours. For the induction of V_H_H display, the medium was exchanged to YNB-CAA medium containing 2% (w/v) galactose with an initial cell optical density at 600 nm (OD_600_) of 0.5. Cells were incubated for 18 to 24 hours at 220 rpm and 30°C. To prepare yeast cells for flow cytometry, cultures were centrifuged at 8000*g* for 1 min. The cells were washed once by resuspending the pellet in PBSA [PBS with bovine serum albumin (1 g/liter)], followed by another centrifugation step. Biotinylated or AF488-labeled toxins were diluted to 100 nM in PBSA, and yeast cells were resuspended at 100 μl of toxin solution per 1 OD_600_ unit (i.e., 100 μl/OD). For polyspecific binding sorts, 100 nM of each toxin was used. The cells were incubated with the toxins for 30 min at 4°C with shaking at 500 rpm. After incubation, the cells were washed with PBSA and resuspended in a staining mix containing either streptavidin-phycoerythrin (PE) (1 μg/ml; 405204, BioLegend) or 500 nM GFP-SpyCatcher. The cells were incubated under the same conditions as before, washed, and resuspended at 1 ml of PBSA per 1 OD_600_ unit for FACS. FACS was performed using a Sony MA900. For cLNTx binding/nonbinding sorts, cells were gated on the basis of toxin binding (PE signal): cLNTx binders were PE+, and cLNTx nonbinders were PE−. In the initial library, nonbinding cells displayed high SpyC-GFP signal, whereas cLNTx-binding cells (PE+) showed notably lower SpyC-GFP signal. We sorted on PE+ signal alone, and across successive sorting rounds, enriched binder populations progressively increased in SpyC-GFP signal intensity, confirming selection for V_H_Hs that both bind cLNTx and display efficiently. For polyspecific sorts using both αBgtx (PE) and αCbtx (AF488), cells were selected on the basis of simultaneous toxin binding signals (PE+ and AF488+), as SpyC-GFP detection could not be simultaneously used with the AF488 fluorophore. After sorting, cells were transferred to SD-UT media and grown for 1 to 2 days until saturation. Sorting was repeated two or three times to achieve enriched populations.

### Illumina deep sequencing

Plasmids from yeast display selections were isolated using the Zymoprep Yeast Plasmid Miniprep II (D2004, Zymo Research). PCR 1 was performed using primers with Nextera adapters and the KAPA HiFi HotStart ReadyMix PCR Kit (KK2601, Roche). Primers were as follows: ngs_pcr1_fwd: 5′-TCGTCGGCAGCGTCAGATGTGTATAAGAGACAGCAGGTGCAGCTGCAGGAG-3′; ngs_pcr1_rev_2: 5′-GTCTCGTGGGCTCGGAGATGTGTATAAGAGACAGAGCGGCCGCTGAGGAG-3′.

PCR products were purified using SPRIselect beads (B23318, Beckman Coulter). PCR 2 was performed using Nextera XT Index Kit v2 Set D (FC-131–2004, Illumina), and PCR products were purified again using SPRIselect beads. Quality control was performed using the Qubit dsDNA HS Assay Kit (Q32854, Thermo Fisher Scientific) and the Agilent High Sensitivity DNA Kit (5067-4626, Agilent). Sequencing was done using the MiSeq Reagent Kit v2, 500 Cycles (MS-102-2003, Illumina) for paired-end sequencing and spiked with 35% PhiX.

### Processing and clustering of sequencing data

Raw sequencing reads were processed using the MiXCR software package (*52*) with the following settings: Paired-end B cell antigen receptor amplicon analysis was performed using mixcr analyze generic-bcr-amplicon in DNA mode (--dna) with the *Lama glama* reference (--species lamaGlama). Rigid left and right alignment boundaries were enforced (--rigid-left-alignment-boundary and --rigid-right-alignment-boundary), and clonotypes were assembled across the full V-region span from FR1Begin to FR4End (--assemble-clonotypes-by “{FR1Begin:FR4End}”). To prioritize sequences supported by each selection, we performed an enrichment-based downselection within paired sorted populations for each screening strategy (cLNTx binder versus nonbinder, cLNTx/αCbtx poly- versus monospecific, and αBgtx/αCbtx poly- versus monospecific). For each pair, we computed each sequence’s relative read fraction within each sample and retained sequences whose fraction was higher in the population of interest than in the paired comparator (i.e., positively enriched), discarding sequences depleted in that comparison. For each selection output, retained sequences were clustered using hierarchical clustering on the basis of pairwise distances computed from global alignments of concatenated CDR1-CDR3 sequences. Duplicate CDR1-CDR3 sequences were removed within each sample before distance calculation. Hierarchical clustering was performed using Ward linkage, and clusters were defined by cutting the dendrogram at a fixed distance threshold. Dendrogram cut thresholds were set empirically to obtain a tractable number of clusters for downstream analysis: 35 for cLNTx binding/nonbinding, 15 for cLNTx/αCbtx poly/monospecific, and 10 for αBgtx/αCbtx poly/monospecific. Two-dimensional visualizations were generated using *t*-distributed stochastic neighbor embedding (*t*-SNE) with n_components = 2, perplexity = 30, and n_iter = 300, applied to the same distance matrix.

### AF3 prediction and evaluation

V_H_H-toxin complex predictions were performed using AF3 implementation (*37*) on the AlphaFold Server (https://alphafoldserver.com) using one seed. To assess prediction reproducibility, a subset of V_H_H-toxin pairs was repredicted using a local AF3 implementation (version 3.0.0) with five independent random seeds (one matching the server prediction for comparison). For each prediction, the best-ranked model was selected, and the ipTM value was extracted. Data were labeled as “predicted binding” if the ipTM value exceeded 0.8. For benchmarking against yeast display selections, “true binding” labels were derived from the corresponding FACS-sorted populations (binder versus nonbinder and mono- versus polyspecific). For experimental validation, “true binding” was defined as split-luciferase log_2_FC > 1. Epitopes were defined as antigen residues within a distance of <4 Å to the V_H_H structure. Epitope analysis was conducted using custom Python scripts with biopython/1.84. Complex structures were visualized using ChimeraX-1.6.1.

### Structural evolution

A structure-informed language model (*38*) was used to propose single-point mutations to potentially improve the affinity of the 19 αBgtx/αCbtx polyspecific V_H_Hs for which high-confidence structures were available. For each V_H_H, the V_H_H-αCbtx and V_H_H-αBgtx complex structures were provided as input to the model. Proposed mutations were then filtered to retain only those suggested for both complexes for a given V_H_H to prioritize variants that enhance binding without disrupting polyspecificity.

### CamSol

The CamSol method (*39*) was used via the online server (www-cohsoftware.ch.cam.ac.uk/index.php/camsolcombination) to propose solubility-improving mutations for the 19 αBgtx/αCbtx polyspecific V_H_Hs with high-confidence structures. To avoid compromising antigen recognition, residues belonging to the paratope were excluded from the set of positions eligible for mutation, restricting design to nonparatope sites expected to improve solubility without directly perturbing the binding interface.

### Split-luciferase screening of binding and expression

The genes encoding a subset of the discovered V_H_Hs and optimized variants were ordered from Twist as OligoPools and assembled by Golden Gate cloning using Bsa I sites. Each V_H_H was fused C-terminally to LargeBiT luciferase, enabling rapid assessment of both expression and antigen binding via a split-luciferase assay (NanoBiT/HiBiT format), conceptually similar to prior implementations (*42*). Constructs were transformed into SHuffle T7 Express *E. coli* cells (C3029J, NEB) and expressed by 1 mM IPTG induction. Cells were lysed, and split-luciferase readouts were performed directly on 1:400 diluted lysates. For expression quantification, lysates were incubated with HiBiT detection reagent for 1 hour, followed by addition of the luciferase substrate furimazine and measurement of luminescence on a plate reader. For binding measurements, lysates were incubated with biotinylated target antigen for 1 hour and then incubated for an additional 1 hour with Lumit streptavidin-SmBiT (W1671, Promega), followed by substrate addition and luminescence measurement. Binding was called positive when the split-luciferase binding readout exceeded the background by log_2_FC > 1, where the background was defined as the luminescence measured for the same V_H_H-LargeBiT lysate incubated without antigen and without SmBiT (V_H_H-LargeBiT + substrate only).

### Expression and purification of selected V_H_Hs

A selection of V_H_Hs was expressed in small-scale cultures for subsequent purification. Expression was initiated by diluting 40 μl of a saturated overnight culture into 4 ml of 2xYT medium, followed by incubation at 37°C with shaking at 250 rpm for 2 hours. Induction was achieved by adding IPTG to a final concentration of 1 mM, and cultures were grown overnight at 25°C with shaking at 250 rpm. Cells were harvested by centrifugation at 4800*g* for 20 min, and pellets were resuspended in 400 μl of PBS (pH 8.0). Cells were lysed by sonication and clarified by centrifugation at 4800*g* for 20 min. The soluble fraction was purified using HisPur Ni-NTA Magnetic Beads (88832, Thermo Fisher Scientific) according to the manufacturer’s workflow using PBS (pH 8.0) as equilibration/wash buffer and PBS (pH 8.0) supplemented with 200 mM imidazole for elution. Dialysis to remove imidazole was performed using 3.5-kDa-molecular-weight-cutoff Pierce Microdialysis Plates (A50467, Thermo Fisher Scientific).

### Binding analysis using BLI

Binding kinetics between purified V_H_Hs and their cognate toxin targets were measured by BLI using an Octet RED96 instrument (ForteBio) at 30°C. All measurements were performed in Hepes buffer (pH 7.2), and sensors were preincubated in Hepes buffer for 30 min. Biotinylated αBgtx and αCbtx (200 nM final) were immobilized on streptavidin biosensors (Sartorius) to a loading response of ∼1.0 nm. To identify an appropriate analyte concentration for the WT V_H_H, toxin-loaded biosensors were exposed to a four-point dilution series of V_H_H (300, 100, 33, and 11 nM). For subsequent measurements comparing WT and optimized variants, the V_H_H concentration was fixed at 33 nM. Association was monitored for 600 s followed by dissociation for 600 s in buffer. Data were reference subtracted using a toxin-loaded sensor incubated without V_H_H. Curves were analyzed in Octet Analysis Studio version 12.2.2.26 (ForteBio) and with custom Python scripts.

### α7nAChR-blocking assay

Dose-response inhibition was assessed by DELFIA (dissociation-enhanced lanthanide fluorescent immunoassay) using a receptor-blocking assay adapted from Ratanabanangkoon *et al.* (*53*). Black MaxiSorp plates (Nunc) were coated overnight at 4°C with human α7nAChR (5 μg/ml) (*54*) in PBS (100 μl per well), then washed three times with PBS-T [PBS with 0.1% (v/v) Tween 20] and blocked with 3% nonfat milk (PanReac AppliChem) in PBS (MPBS). Threefold serial dilutions of V_H_Hs (starting at 200 nM) were mixed 1:1 (30 μl each) with biotinylated αBgtx (0.2 μg/ml) or αCbtx (0.2 μg/ml) in MPBS and incubated for 30 min at room temperature. Mixtures were transferred to blocked wells and incubated for 1 hour. After three washes with PBS-T, bound toxin was detected with a 1:500 dilution of europium-labeled streptavidin (Revvity) in DELFIA assay buffer (Revvity) for 30 min (50 μl per well), followed by DELFIA Enhancement Solution (50 μl per well). Fluorescence was measured on a VICTOR Nivo multimode plate reader (ex. 320 nm/em. 615 nm). All samples were run in triplicate. Data were analyzed using custom Python scripts.

## References

[1] X. Lyu, Q. Zhao, J. Hui, T. Wang, M. Lin, K. Wang, J. Zhang, J. Shentu, P. A. Dalby, H. Zhang, B. Liu, The global landscape of approved antibody therapies. Antib. Ther. 5, 233–257 (2022). 10.1093/abt/tbac021 PMC9535261 36213257

[2] T. T. Khuat, R. Bassett, E. Otte, A. Grevis-James, B. Gabrys, Applications of machine learning in antibody discovery, process development, manufacturing and formulation: Current trends, challenges, and opportunities. Comput. Chem. Eng. 182, 108585 (2024).

[3] M. L. Fernández-Quintero, A. Ljungars, F. Waibl, V. Greiff, J. T. Andersen, T. T. Gjølberg, T. P. Jenkins, B. G. Voldborg, L. M. Grav, S. Kumar, G. Georges, H. Kettenberger, K. R. Liedl, P. M. Tessier, J. McCafferty, A. H. Laustsen, Assessing developability early in the discovery process for novel biologics. mAbs 15, 2171248 (2023). 10.1080/19420862.2023.2171248 PMC9980699 36823021

[4] L. Li, E. Gupta, J. Spaeth, L. Shing, R. Jaimes, E. Engelhart, R. Lopez, R. S. Caceres, T. Bepler, M. E. Walsh, Machine learning optimization of candidate antibody yields highly diverse sub-nanomolar affinity antibody libraries. Nat. Commun. 14, 3454 (2023). 10.1038/s41467-023-39022-2 PMC10258481 37308471

[5] A. H. Laustsen, V. Greiff, A. Karatt-Vellatt, S. Muyldermans, T. P. Jenkins, Animal immunization, in vitro display technologies, and machine learning for antibody discovery. Trends Biotechnol. 39, 1263–1273 (2021). 10.1016/j.tibtech.2021.03.003 33775449

[6] Z.-J. Lu, S.-J. Deng, D.-G. Huang, Y. He, M. Lei, L. Zhou, P. Jin, Frontier of therapeutic antibody discovery: The challenges and how to face them. World J. Biol. Chem. 3, 187–196 (2012). 10.4331/wjbc.v3.i12.187 PMC3531614 23275803

[7] N. L. S. Roberts, E. K. Johnson, S. M. Zeng, E. B. Hamilton, A. Abdoli, F. Alahdab, V. Alipour, R. Ancuceanu, C. L. Andrei, D. Anvari, J. Arabloo, M. Ausloos, A. F. Awedew, A. D. Badiye, S. M. Bakkannavar, A. Bhalla, N. Bhardwaj, P. Bhardwaj, S. Bhaumik, A. Bijani, A. Boloor, T. Cai, F. Carvalho, D.-T. Chu, R. A. S. Couto, X. Dai, A. A. Desta, H. T. Do, L. Earl, A. Eftekhari, F. Esmaeilzadeh, F. Farzadfar, E. Fernandes, I. Filip, M. Foroutan, R. C. Franklin, A. M. Gaidhane, B. G. Gebregiorgis, B. Gebremichael, A. Ghashghaee, M. Golechha, S. Hamidi, S. E. Haque, K. Hayat, C. Herteliu, O. S. Ilesanmi, M. M. Islam, J. Jagnoor, T. Kanchan, N. Kapoor, E. A. Khan, M. N. Khatib, R. Khundkar, K. Krishan, G. A. Kumar, N. Kumar, I. Landires, S. S. Lim, M. Madadin, V. Maled, N. Manafi, L. B. Marczak, R. G. Menezes, T. J. Meretoja, T. R. Miller, A. Mohammadian-Hafshejani, A. H. Mokdad, F. N. P. Monteiro, M. Moradi, V. C. Nayak, C. T. Nguyen, H. L. T. Nguyen, V. Nuñez-Samudio, S. M. Ostroff, J. R. Padubidri, H. Q. Pham, M. Pinheiro, M. Pirestani, Z. Quazi Syed, N. Rabiee, A. Radfar, V. Rahimi-Movaghar, S. J. Rao, P. Rastogi, D. L. Rawaf, S. Rawaf, R. C. Reiner, A. Sahebkar, A. M. Samy, M. Sawhney, D. C. Schwebel, S. Senthilkumaran, M. A. Shaikh, V. Y. Skryabin, A. A. Skryabina, A. Soheili, M. A. Stokes, R. Thapar, M. R. Tovani-Palone, B. X. Tran, R. S. Travillian, D. Z. Velazquez, Z.-J. Zhang, M. Naghavi, R. Dandona, L. Dandona, S. L. James, D. M. Pigott, C. J. L. Murray, S. I. Hay, T. Vos, K. L. Ong, GBD, Snakebite envenomation collaborators, global mortality of snakebite envenoming between 1990 and 2019. Nat. Commun. 13, 6160 (2022). 10.1038/s41467-022-33627-9 PMC9596405 36284094

[8] A. H. Laustsen, K. H. Johansen, M. Engmark, M. R. Andersen, Recombinant snakebite antivenoms: A cost-competitive solution to a neglected tropical disease? PLoS Negl. Trop. Dis. 11, e0005361 (2017). 10.1371/journal.pntd.0005361 PMC5310919 28158193

[9] S. Ahmadi, N. J. Burlet, M. Benard-Valle, A. Guadarrama-Martínez, S. Kerwin, I. A. Cardoso, A. E. Marriott, R. J. Edge, E. Crittenden, E. Neri-Castro, M. L. Fernandez-Quintero, G. T. T. Nguyen, C. O’Brien, Y. Wouters, K. Kalogeropoulos, S. Thumtecho, T. W. Ebersole, C. H. Dahl, E. U. Glegg-Sørensen, T. Jansen, K. Boddum, E. Manousaki, E. Rivera-de-Torre, A. B. Ward, J. P. Morth, A. Alagón, S. P. Mackessy, S. Ainsworth, S. K. Menzies, N. R. Casewell, T. P. Jenkins, A. Ljungars, A. H. Laustsen, Nanobody-based recombinant antivenom for cobra, mamba and rinkhals bites. Nature 647, 716–725 (2025). 10.1038/s41586-025-09661-0 PMC12629983 41162699

[10] L. Ledsgaard, J. Wade, T. P. Jenkins, K. Boddum, I. Oganesyan, J. A. Harrison, P. Villar, R. A. Leah, R. Zenobi, S. Schoffelen, B. Voldborg, A. Ljungars, J. McCafferty, B. Lomonte, J. M. Gutiérrez, A. H. Laustsen, A. Karatt-Vellatt, Discovery and optimization of a broadly-neutralizing human monoclonal antibody against long-chain α-neurotoxins from snakes. Nat. Commun. 14, 682 (2023). 10.1038/s41467-023-36393-4 PMC9908967 36755049

[11] I. S. Khalek, R. R. Senji Laxme, Y. T. K. Nguyen, S. Khochare, R. N. Patel, J. Woehl, J. M. Smith, K. Saye-Francisco, Y. Kim, L. Misson Mindrebo, Q. Tran, M. Kędzior, E. Boré, O. Limbo, M. Verma, R. L. Stanfield, S. K. Menzies, S. Ainsworth, R. A. Harrison, D. R. Burton, D. Sok, I. A. Wilson, N. R. Casewell, K. Sunagar, J. G. Jardine, Synthetic development of a broadly neutralizing antibody against snake venom long-chain α-neurotoxins. Sci. Transl. Med. 16, eadk1867 (2024). 10.1126/scitranslmed.adk1867 PMC7617732 38381847

[12] M. Benard-Valle, Y. Wouters, A. Ljungars, G. T. T. Nguyen, S. Ahmadi, T. W. Ebersole, C. H. Dahl, A. Guadarrama-Martínez, F. Jeppesen, H. Eriksen, G. Rodríguez-Barrera, K. Boddum, T. P. Jenkins, S. P. Bjørn, S. Schoffelen, B. G. Voldborg, A. Alagón, A. H. Laustsen, In vivo neutralization of coral snake venoms with an oligoclonal nanobody mixture in a murine challenge model. Nat. Commun. 15, 4310 (2024). 10.1038/s41467-024-48539-z PMC11109316 38773068

[13] A. H. Laustsen, A. Karatt-Vellatt, E. W. Masters, A. S. Arias, U. Pus, C. Knudsen, S. Oscoz, P. Slavny, D. T. Griffiths, A. M. Luther, R. A. Leah, M. Lindholm, B. Lomonte, J. M. Gutiérrez, J. McCafferty, In vivo neutralization of dendrotoxin-mediated neurotoxicity of black mamba venom by oligoclonal human IgG antibodies. Nat. Commun. 9, 3928 (2018). 10.1038/s41467-018-06086-4 PMC6168529 30279409

[14] A. H. Laustsen, J. María Gutiérrez, C. Knudsen, K. H. Johansen, E. Bermúdez-Méndez, F. A. Cerni, J. A. Jürgensen, L. Ledsgaard, A. Martos-Esteban, M. Øhlenschlæger, U. Pus, M. R. Andersen, B. Lomonte, M. Engmark, M. B. Pucca, Pros and cons of different therapeutic antibody formats for recombinant antivenom development. Toxicon 146, 151–175 (2018). 10.1016/j.toxicon.2018.03.004 29534892

[15] T. P. Jenkins, A. H. Laustsen, Cost of manufacturing for recombinant snakebite antivenoms. Front. Bioeng. Biotechnol. 8, 703 (2020). 10.3389/fbioe.2020.00703 PMC7381290 32766215

[16] A. H. Laustsen, M. Benard-Valle, A. G. Habib, N. R. Casewell, M. Abouyannis, D. G. Lalloo, A. Ljungars, Target product profiles for pan-Africa recombinant antivenoms against neurotoxic or hemotoxic and cytotoxic snakebite envenoming. PLoS Negl. Trop. Dis. 19, e0012833 (2025). 10.1371/journal.pntd.0012833 PMC11759373 39854596

[17] A. H. Laustsen, B. Lomonte, B. Lohse, J. Fernández, J. M. Gutiérrez, Unveiling the nature of black mamba (*Dendroaspis polylepis*) venom through venomics and antivenom immunoprofiling: Identification of key toxin targets for antivenom development. J. Proteomics 119, 126–142 (2015). 10.1016/j.jprot.2015.02.002 25688917

[18] A. H. Laustsen, J. M. Gutiérrez, B. Lohse, A. R. Rasmussen, J. Fernández, C. Milbo, B. Lomonte, Snake venomics of monocled cobra (*Naja kaouthia*) and investigation of human IgG response against venom toxins. Toxicon Off. J. Int. Soc. Toxinology 99, 23–35 (2015). 10.1016/j.toxicon.2015.03.001 25771242

[19] P. Kessler, P. Marchot, M. Silva, D. Servent, The three-finger toxin fold: A multifunctional structural scaffold able to modulate cholinergic functions. J. Neurochem. 142, 7–18 (2017). 10.1111/jnc.13975 28326549

[20] D. A. Warrell, “Clinical toxicology of snakebite in Asia” in *Handbook of Clinical Toxicology of Animal Venoms and Poisons* (CRC Press, 2008).

[21] S. Miersch, G. de la Rosa, R. Friis, L. Ledsgaard, K. Boddum, A. H. Laustsen, S. S. Sidhu, Synthetic antibodies block receptor binding and current-inhibiting effects of α-cobratoxin from *Naja kaouthia*. Protein Sci. 31, e4296 (2022). 10.1002/pro.4296 PMC8994502 35481650

[22] G. Richard, A. J. Meyers, M. D. McLean, M. Arbabi-Ghahroudi, R. MacKenzie, J. C. Hall, In vivo neutralization of α-cobratoxin with high-affinity llama single-domain antibodies (VHHs) and a VHH-Fc antibody. PLOS ONE 8, e69495 (2013). 10.1371/journal.pone.0069495 PMC3718736 23894495

[23] S. Vázquez Torres, M. Benard Valle, S. P. Mackessy, S. K. Menzies, N. R. Casewell, S. Ahmadi, N. J. Burlet, E. Muratspahić, I. Sappington, M. D. Overath, E. Rivera-de-Torre, J. Ledergerber, A. H. Laustsen, K. Boddum, A. K. Bera, A. Kang, E. Brackenbrough, I. A. Cardoso, E. P. Crittenden, R. J. Edge, J. Decarreau, R. J. Ragotte, A. S. Pillai, M. Abedi, H. L. Han, S. R. Gerben, A. Murray, R. Skotheim, L. Stuart, L. Stewart, T. J. A. Fryer, T. P. Jenkins, D. Baker, De novo designed proteins neutralize lethal snake venom toxins. Nature 639, 225–231 (2025). 10.1038/s41586-024-08393-x PMC11882462 39814879

[24] A. Ljungars, A. H. Laustsen, Neutralization capacity of recombinant antivenoms based on monoclonal antibodies and nanobodies. Toxicon 222, 106991 (2023). 10.1016/j.toxicon.2022.106991 36481349

[25] P. Stargardt, A. Ljungars, D. Scheich, N. Lingg, E. S. Elmose, S. Ahmadi, F. Simon, A. Jungbauer, J. Mairhofer, A. H. Laustsen, One-pot expression of an oligoclonal nanobody-based antivenom in growth-decoupled *Escherichia coli*. Trends Biotechnol., S0167-7799(26)00085-5 (2026). 10.1016/j.tibtech.2026.02.022 42062180

[26] F. Ferrara, L. A. Naranjo, S. Kumar, T. Gaiotto, H. Mukundan, B. Swanson, A. R. M. Bradbury, Using phage and yeast display to select hundreds of monoclonal antibodies: Application to antigen 85, a tuberculosis biomarker. PLOS ONE 7, e49535 (2012). 10.1371/journal.pone.0049535 PMC3498134 23166701

[27] F. Ferrara, M. F. Soluri, D. Sblattero, “Recombinant antibody selections by combining phage and yeast display” in *Human Monoclonal Antibodies: Methods and Protocols*, M. Steinitz, Ed. (Springer, New York, NY, 2019), pp. 339–352; 10.1007/978-1-4939-8958-4_16. 30539479

[28] Y. Sun, B. Ban, A. Bradbury, G. A. S. Ansari, D. A. Blake, Combining yeast display and competitive FACS to select rare hapten-specific clones from recombinant antibody libraries. Anal. Chem. 88, 9181–9189 (2016). 10.1021/acs.analchem.6b02334 PMC5032104 27571429

[29] V. Greiff, E. Miho, U. Menzel, S. T. Reddy, Bioinformatic and statistical analysis of adaptive immune repertoires. Trends Immunol. 36, 738–749 (2015). 10.1016/j.it.2015.09.006 26508293

[30] K. Barreto, B. V. Maruthachalam, W. Hill, D. Hogan, A. R. Sutherland, A. Kusalik, H. Fonge, J. F. DeCoteau, C. R. Geyer, Next-generation sequencing-guided identification and reconstruction of antibody CDR combinations from phage selection outputs. Nucleic Acids Res. 47, e50 (2019). 10.1093/nar/gkz131 PMC6511873 30854567

[31] S. Lu, A. K. Mattox, P. Aitana Azurmendi, I. Christodoulou, K. M. Wright, M. Popoli, Z. Chen, S. Sur, Y. Li, C. L. Bonifant, C. Bettegowda, N. Papadopoulos, S. Zhou, S. B. Gabelli, B. Vogelstein, K. W. Kinzler, The rapid and highly parallel identification of antibodies with defined biological activities by SLISY. Nat. Commun. 14, 17 (2023). 10.1038/s41467-022-35668-6 PMC9808734 36596784

[32] D. M. Mason, S. Friedensohn, C. R. Weber, C. Jordi, B. Wagner, S. M. Meng, R. A. Ehling, L. Bonati, J. Dahinden, P. Gainza, B. E. Correia, S. T. Reddy, Optimization of therapeutic antibodies by predicting antigen specificity from antibody sequence via deep learning. Nat. Biomed. Eng. 5, 600–612 (2021). 10.1038/s41551-021-00699-9 33859386

[33] Y. W. Lim, A. S. Adler, D. S. Johnson, Predicting antibody binders and generating synthetic antibodies using deep learning. MAbs 14, 2069075 (2022). 10.1080/19420862.2022.2069075 PMC9067455 35482911

[34] E. K. Makowski, P. C. Kinnunen, J. Huang, L. Wu, M. D. Smith, T. Wang, A. A. Desai, C. N. Streu, Y. Zhang, J. M. Zupancic, J. S. Schardt, J. J. Linderman, P. M. Tessier, Co-optimization of therapeutic antibody affinity and specificity using machine learning models that generalize to novel mutational space. Nat. Commun. 13, 3788 (2022). 10.1038/s41467-022-31457-3 PMC9249733 35778381

[35] P. Arras, H. B. Yoo, L. Pekar, T. Clarke, L. Friedrich, C. Schröter, J. Schanz, J. Tonillo, V. Siegmund, A. Doerner, S. Krah, E. Guarnera, S. Zielonka, A. Evers, AI/ML combined with next-generation sequencing of VHH immune repertoires enables the rapid identification of de novo humanized and sequence-optimized single domain antibodies: A prospective case study. Front. Mol. Biosci. 10, 1249247 (2023). 10.3389/fmolb.2023.1249247 PMC10575757 37842638

[36] R. A. Ehling, M. Minot, M. D. Overath, D. J. Sheward, J. Han, B. Gao, J. M. Taft, M. Pertseva, C. R. Weber, L. Frei, T. Bikias, B. Murrell, S. T. Reddy, Synthetic coevolution reveals adaptive mutational trajectories of neutralizing antibodies and SARS-CoV-2. Cell Syst. 16, 101391 (2025). 10.1016/j.cels.2025.101391 40967184

[37] J. Abramson, J. Adler, J. Dunger, R. Evans, T. Green, A. Pritzel, O. Ronneberger, L. Willmore, A. J. Ballard, J. Bambrick, S. W. Bodenstein, D. A. Evans, C.-C. Hung, M. O’Neill, D. Reiman, K. Tunyasuvunakool, Z. Wu, A. Žemgulytė, E. Arvaniti, C. Beattie, O. Bertolli, A. Bridgland, A. Cherepanov, M. Congreve, A. I. Cowen-Rivers, A. Cowie, M. Figurnov, F. B. Fuchs, H. Gladman, R. Jain, Y. A. Khan, C. M. R. Low, K. Perlin, A. Potapenko, P. Savy, S. Singh, A. Stecula, A. Thillaisundaram, C. Tong, S. Yakneen, E. D. Zhong, M. Zielinski, A. Žídek, V. Bapst, P. Kohli, M. Jaderberg, D. Hassabis, J. M. Jumper, Accurate structure prediction of biomolecular interactions with AlphaFold 3. Nature 630, 493–500 (2024). 10.1038/s41586-024-07487-w PMC11168924 38718835

[38] V. R. Shanker, T. U. J. Bruun, B. L. Hie, P. S. Kim, Unsupervised evolution of protein and antibody complexes with a structure-informed language model. Science 385, 46–53 (2024). 10.1126/science.adk8946 PMC11616794 38963838

[39] P. Sormanni, F. A. Aprile, M. Vendruscolo, The CamSol method of rational design of protein mutants with enhanced solubility. J. Mol. Biol. 427, 478–490 (2015). 10.1016/j.jmb.2014.09.026 25451785

[40] A. Damsbo, N. J. Burlet, M. L. Fernández-Quintero, M. Benard-Valle, M. D. Overath, A. Guadarrama-Martínez, A. Vlamynck, I. Bisbo, C. Villalobos, T. Tulika, A. Alagón, J. R. Loeffler, A. B. Ward, K. Boddum, J. P. Morth, E. Rivera-de-Torre, A. H. Laustsen, Structural mechanisms behind the neutralisation of long-chain α-neurotoxins by broadly neutralising VHHs discovered using a consensus antigen. Commun. Chem. 8, 209 (2025). 10.1038/s42004-025-01600-4 PMC12274480 40681851

[41] EMBL-EBI, AlphaFold: Confidence scores in AlphaFold-Multimer; www.ebi.ac.uk/training/online/courses/alphafold/inputs-and-outputs/evaluating-alphafolds-predicted-structures-using-confidence-scores/confidence-scores-in-alphafold-multimer/.

[42] L. S. Mille-Fragoso, J. N. Wang, C. L. Driscoll, H. Dai, T. Widatalla, X. Zhang, B. L. Hie, X. J. Gao, Efficient generation of epitope-targeted de novo antibodies with Germinal. bioRxiv 677421 [Preprint] (2025); 10.1101/2025.09.19.677421. PMC13366713 42337361

[43] N. R. Bennett, J. L. Watson, R. J. Ragotte, A. J. Borst, D. L. See, C. Weidle, R. Biswas, Y. Yu, E. L. Shrock, R. Ault, P. J. Y. Leung, B. Huang, I. Goreshnik, J. Tam, K. D. Carr, B. Singer, C. Criswell, B. I. M. Wicky, D. Vafeados, M. Garcia Sanchez, H. M. Kim, S. Vázquez Torres, S. Chan, S. M. Sun, T. T. Spear, Y. Sun, K. O’Reilly, J. M. Maris, N. G. Sgourakis, R. A. Melnyk, C. C. Liu, D. Baker, Atomically accurate de novo design of antibodies with RFdiffusion. Nature 649, 183–193 (2026). 10.1038/s41586-025-09721-5 PMC12727541 41193805

[44] Y. Bourne, T. T. Talley, S. B. Hansen, P. Taylor, P. Marchot, Crystal structure of a Cbtx–AChBP complex reveals essential interactions between snake α-neurotoxins and nicotinic receptors. EMBO J. 24, 1512–1522 (2005). 10.1038/sj.emboj.7600620 PMC1142565 15791209

[45] Z. Sang, Y. Xiang, W. Huang, P. R. Sargunas, Y. J. Kim, Z. Feng, D. J. Taylor, Y. Shi, Repertoire-scale antibody structural prediction informs therapeutic design. Sci. Adv. 12, eaef7163 (2026). 10.1126/sciadv.aef7163 PMC13108562 42030385

[46] N. Raouraoua, M. F. Lensink, G. Brysbaert, Massive sampling strategy for antibody–antigen targets in CAPRI Round 55 with MassiveFold. Proteins 2025, 1–9 (2025). 10.1002/prot.26802 PMC13648903 39868877

[47] B. Wallner, AFsample: Improving multimer prediction with AlphaFold using massive sampling. Bioinformatics 39, btad573 (2023). 10.1093/bioinformatics/btad573 PMC10534052 37713472

[48] E. Smorodina, M. Ali, K. K. Brumat, L. Salicari, S. Miklavc, A. Kappassov, C. Fu, P. Sormanni, A. de Marco, V. Greiff, Structural plausibility without binding specificity: Limits of AI-based antibody-antigen structure prediction confidence scores. bioRxiv 709004 [Preprint] (2026); 10.64898/2026.03.02.709004.

[49] Y. Zhang, C. Gong, J. Sun, J. Guan, M. Ren, S. Xue, H. Zhang, W. Ma, Z. Liu, X. Chen, W. Xiao, Protenix-v2: Broadening the reach of structure prediction and biomolecular design. bioRxiv 717613 [Preprint] (2026); 10.64898/2026.04.10.717613.

[50] R. D. Gietz, R. H. Schiestl, High-efficiency yeast transformation using the LiAc/SS carrier DNA/PEG method. Nat. Protoc. 2, 31–34 (2007). 10.1038/nprot.2007.13 17401334

[51] I. N. A. Khairil Anuar, A. Banerjee, A. H. Keeble, A. Carella, G. I. Nikov, M. Howarth, Spy&Go purification of SpyTag-proteins using pseudo-SpyCatcher to access an oligomerization toolbox. Nat. Commun. 10, 1734 (2019). 10.1038/s41467-019-09678-w PMC6465384 30988307

[52] D. A. Bolotin, S. Poslavsky, I. Mitrophanov, M. Shugay, I. Z. Mamedov, E. V. Putintseva, D. M. Chudakov, MiXCR: Software for comprehensive adaptive immunity profiling. Nat. Methods 12, 380–381 (2015). 10.1038/nmeth.3364 25924071

[53] K. Ratanabanangkoon, P. Simsiriwong, K. Pruksaphon, K. Y. Tan, B. Chantrathonkul, S. Eursakun, C. H. Tan, An in vitro potency assay using nicotinic acetylcholine receptor binding works well with antivenoms against *Bungarus candidus* and *Naja naja*. Sci. Rep. 8, 9716 (2018). 10.1038/s41598-018-27794-3 PMC6018763 29946111

[54] L. Ledsgaard, A. H. Laustsen, U. Pus, J. Wade, P. Villar, K. Boddum, P. Slavny, E. W. Masters, A. S. Arias, S. Oscoz, D. T. Griffiths, A. M. Luther, M. Lindholm, R. A. Leah, M. S. Møller, H. Ali, J. McCafferty, B. Lomonte, J. M. Gutiérrez, A. Karatt-Vellatt, In vitro discovery of a human monoclonal antibody that neutralizes lethality of cobra snake venom. MAbs 14, 2085536 (2022). 10.1080/19420862.2022.2085536 PMC9225616 35699567

[55] M. Harel, R. Kasher, A. Nicolas, J. M. Guss, M. Balass, M. Fridkin, A. B. Smit, K. Brejc, T. K. Sixma, E. Katchalski-Katzir, J. L. Sussman, S. Fuchs, The binding site of acetylcholine receptor as visualized in the x-ray structure of a complex between α-bungarotoxin and a mimotope peptide. Neuron 32, 265–275 (2001). 10.1016/s0896-6273(01)00461-5 11683996

