# Supplementary material for: Multiobjective VHH discovery through integrated high-throughput screening and AlphaFold3-guided structural prioritization
Source: Sci Adv. 2026 Sep 30;12(40):eaef5325. doi: 10.1126/sciadv.aef5325 (PMC13626040; doi:10.1126/sciadv.aef5325)
Supplement: Supplementary file 1 [file sciadv.aef5325_sm.pdf]

Science Advances
AAAS

Supplementary Materials for

Multiobjective $V_H H$ discovery through integrated high-throughput screening and AlphaFold3-guided structural prioritization

Max D. Overath *et al.*

Corresponding author: Max D. Overath, maxove@dtu.dk; Timothy P. Jenkins, tpaje@dtu.dk

Sci. Adv. **12**, eaef5325 (2026)
DOI: 10.1126/sciadv.aef5325

The PDF file includes:

Figs. S1 to S13
Legends for data S1 and S2

Other Supplementary Material for this manuscript includes the following:

Data S2

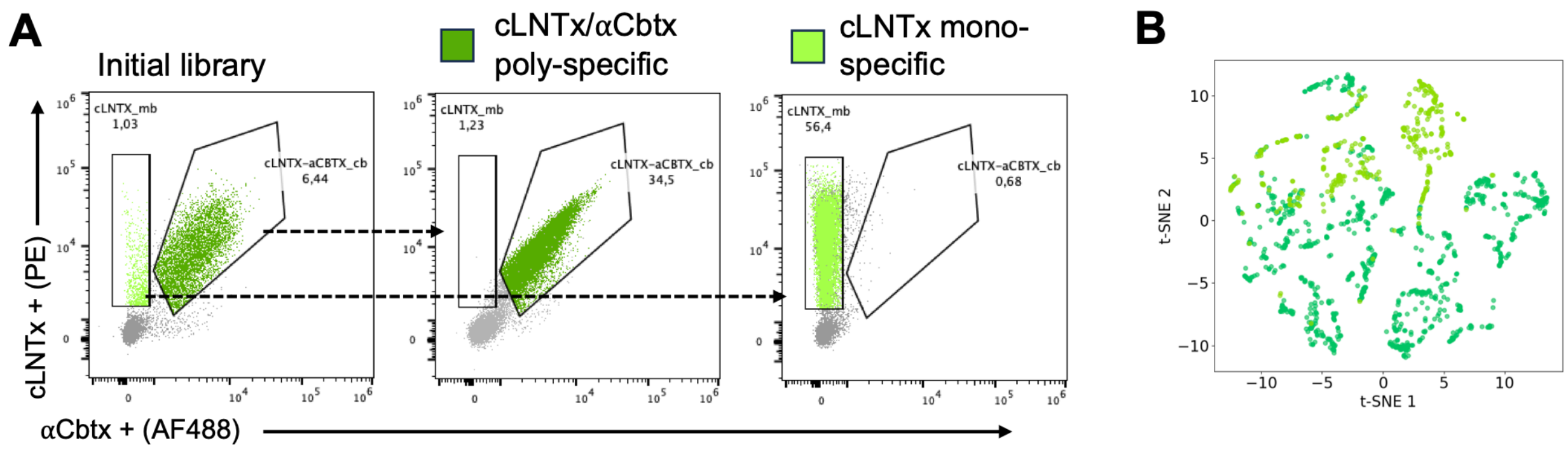

Fig. S1. cLNTx/αCbtx yeast display selections and CDR clustering. a) Poly- and mono-specific yeast-display selections using fluorescently labelled cLNTx and αCBTx as antigens. b) Clustering of CDR sequences for cLNTx/αCBTx poly-specific and cLNTx mono-specific samples**.**

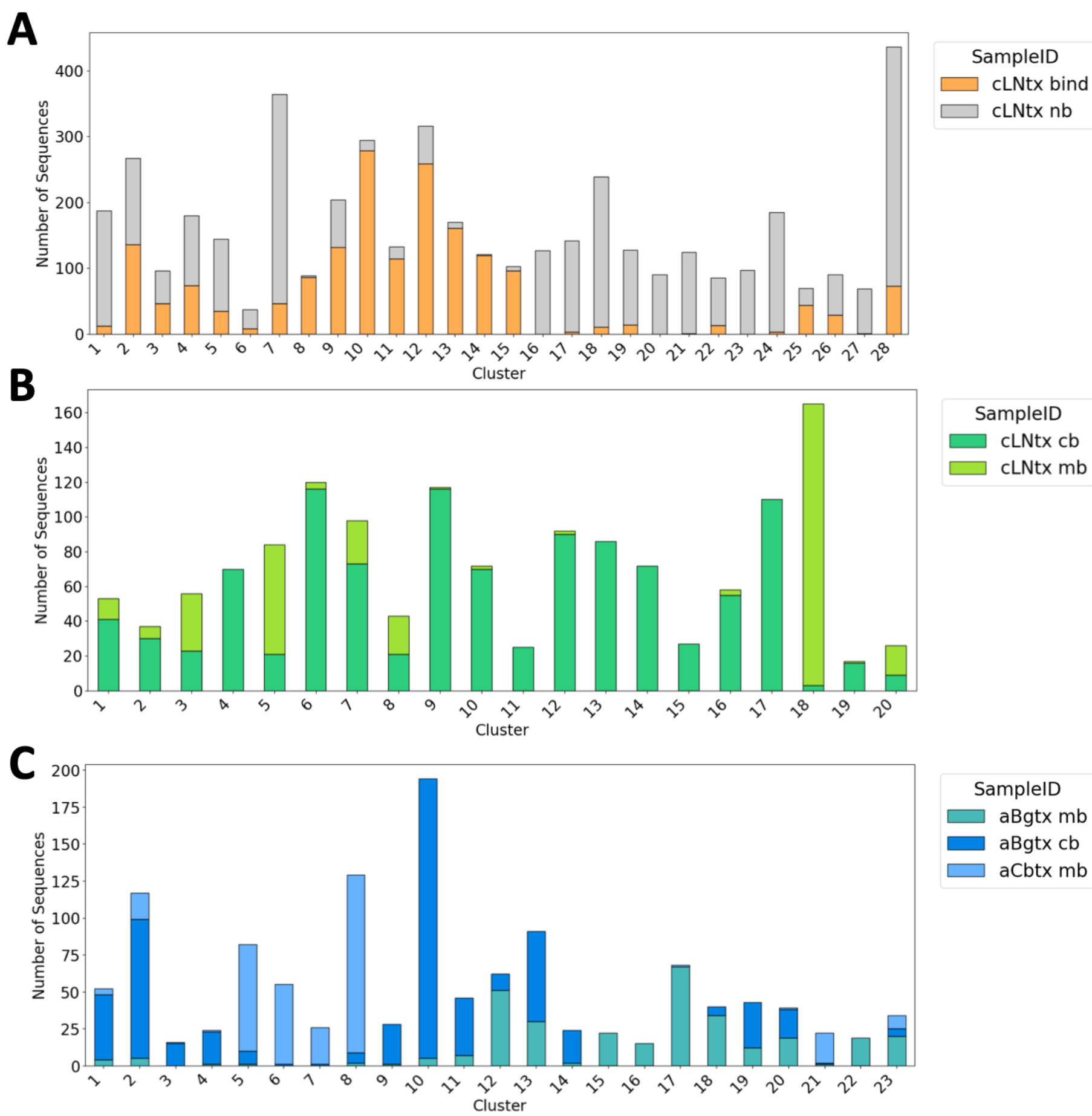

Fig. S2. Distribution of different samples per cluster. CDR sequences were clustered using hierarchical clustering of CDR1-3. The plot shows the frequency of each sample within each cluster, revealing that most clusters consist mainly of a single sample. Data were separated by selection for: a) cLNTx bind and non-bind; b) cLNTx/αCbtx poly-specific and αCbtx mono-specific; and c) αBgtx/αCbtx poly-specific, αCbtx mono-specific, and αBgtx mono-specific.

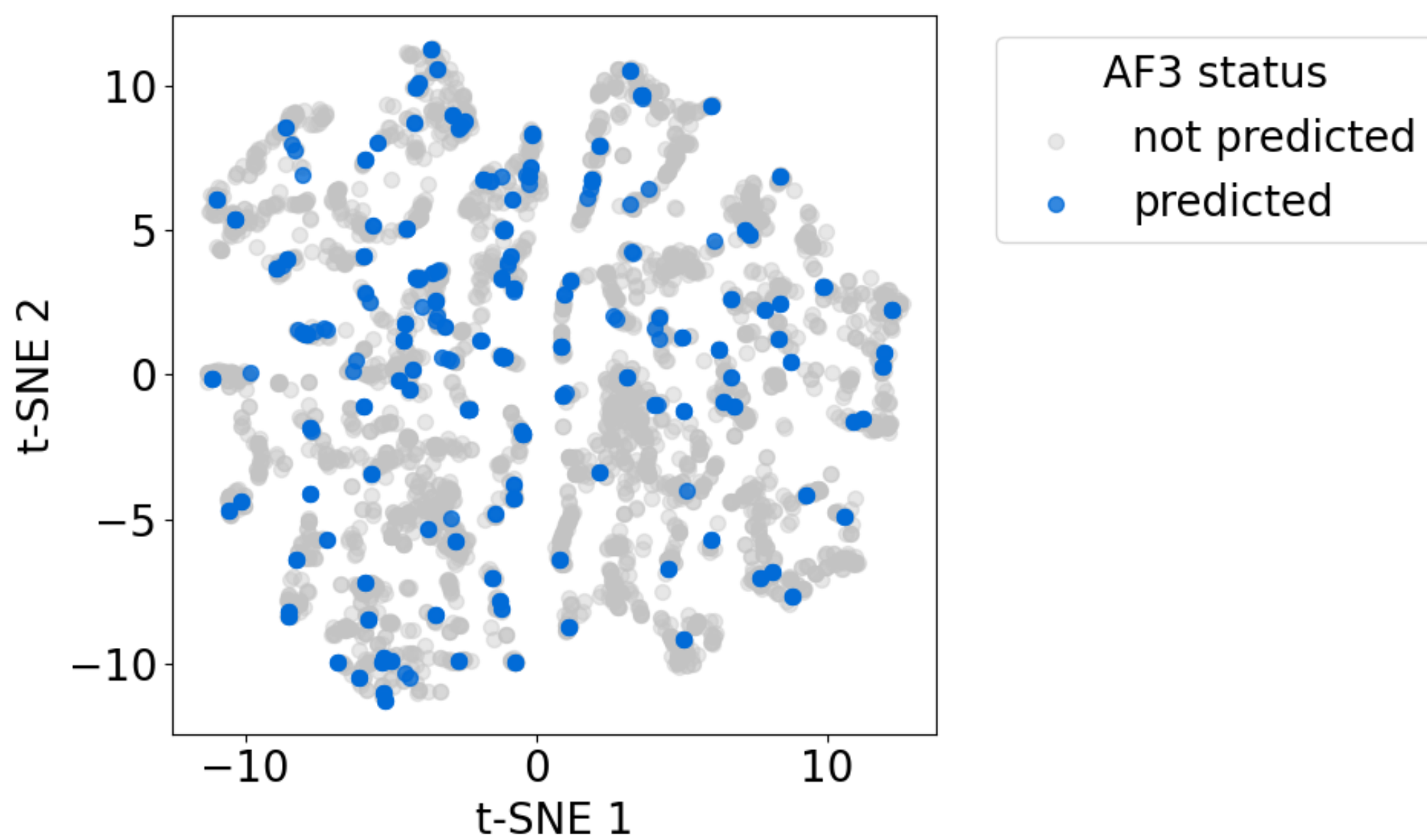

Fig. S3. Coverage of AF3-predicted V_HHs across the deep-sequenced library. t-SNE visualization of CDR1-CDR3 sequence space for all V_HH sequences retained after enrichment-based filtering across selection populations (grey). Blue points indicate sequences selected for AF3 complex prediction. Points are distributed across the full t-SNE landscape, indicating broad sampling of the available sequence diversity.

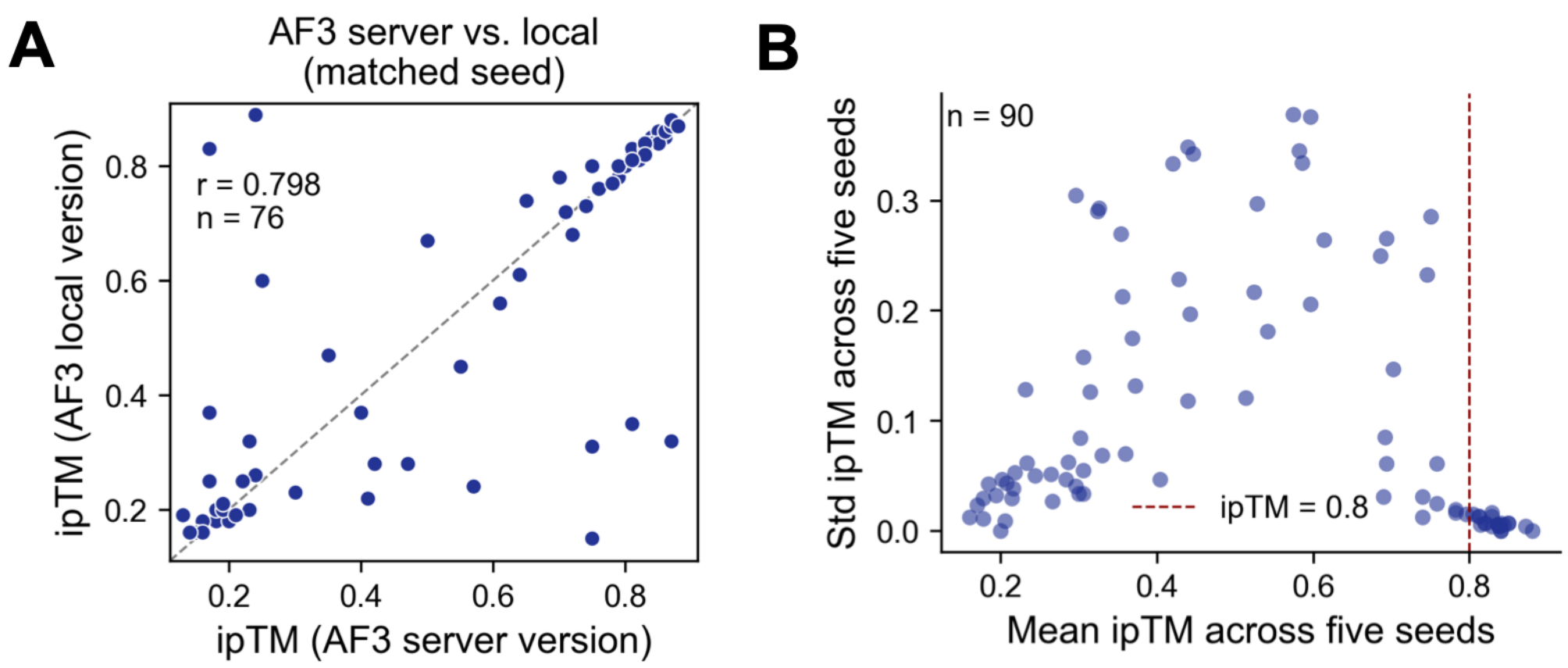

Fig. S4. Reproducibility of AF3 predictions across implementations and random seeds. a) Comparison of ipTM scores for a subset of V_HH-toxin pairs (n = 76) predicted using the AF3 server (used for the main analysis) and a local AF3 implementation (v3.0.0) with a matched random seed. Pearson r = 0.798, indicating overall concordance between implementations, with greatest agreement at low and high confidence scores and more variability in the intermediate range. b) Seed stability analysis of a subset of V_HH-toxin predictions (n = 90) using five independent random seeds on the local AF3 implementation. Each point represents the mean and standard deviation of ipTM across seeds for a single V_HH-toxin pair. Predictions above the ipTM = 0.8 threshold (dashed line) show near-zero variance, confirming that high-confidence predictions are robust to seed choice.

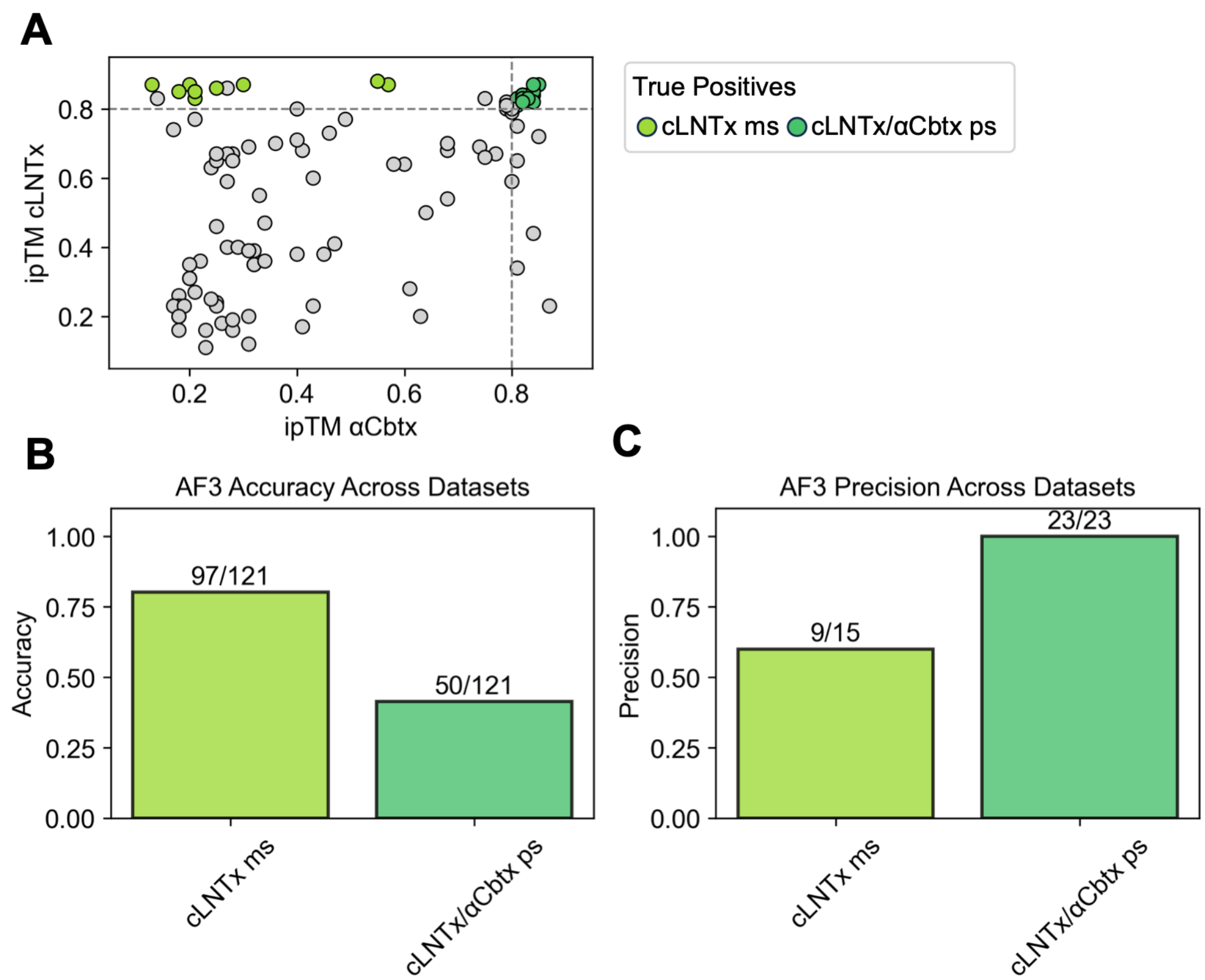

Fig. S5. cLNTx/αCbtx AF3 predictions evaluation. a) ipTM distributions for V_HH-toxin predictions, where colored dots indicate true-positive predictions for cLNTX/αCBTx poly-specific and αCbtx mono-specific samples. b) Accuracy and c) precision plots show very high precision for cLNTX/αCBTx poly-specific V_HHs (precision = 1.0) and lower precision for cLNTx mono-specific samples (precision = 0.6), while accuracy is higher for mono-binding samples (accuracy = 0.80) than for cross-binding samples (accuracy = 0.46).

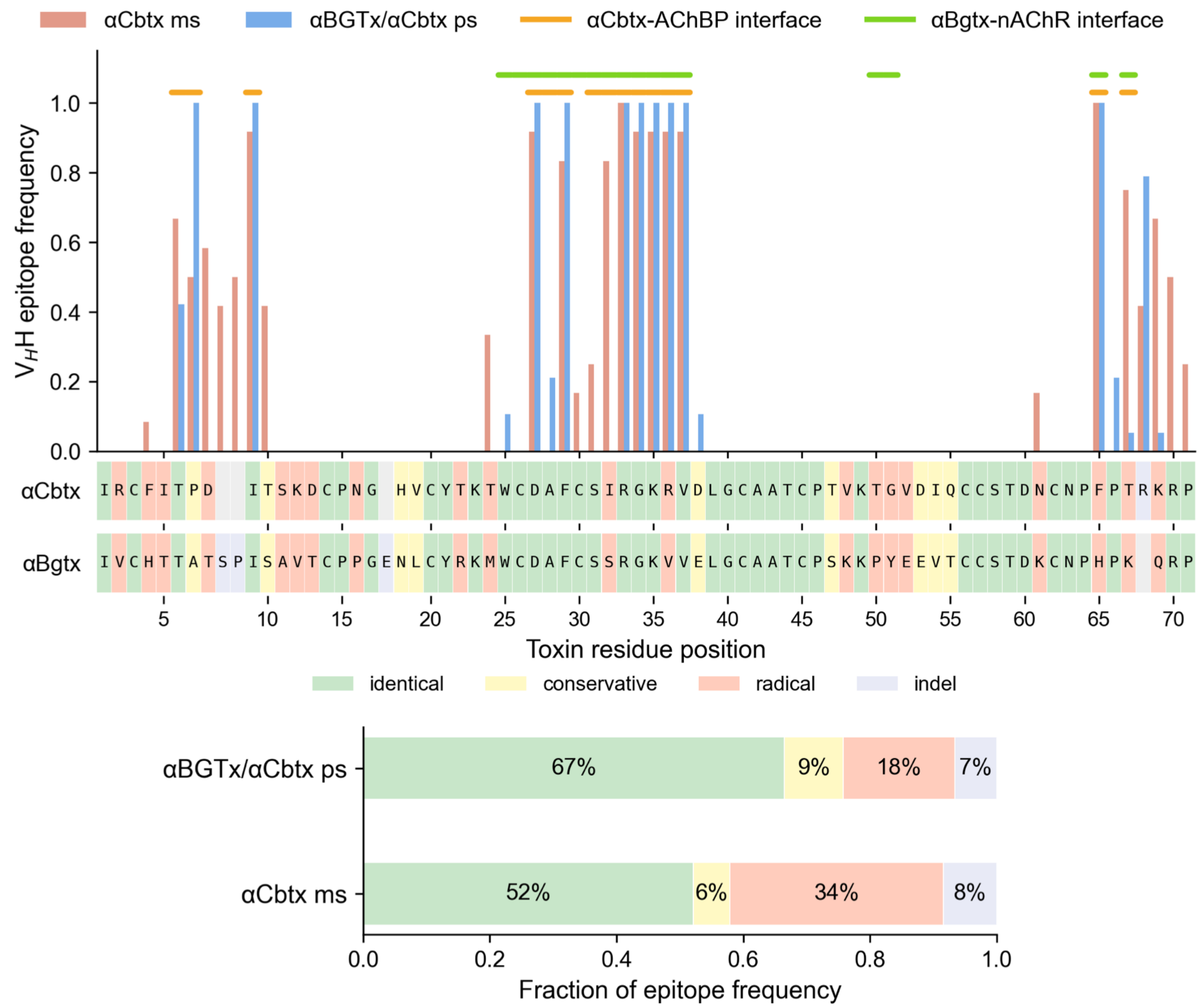

Fig. S6. Sequence conservation and V_HH epitope targeting. V_HH epitope frequency (bars) and sequence alignment of αCbtx and αBgtx. Top: Per-residue epitope frequency for αCbtx mono-specific and αBgtx/αCbtx poly-specific V_HHs. Orange and green markers indicate the αCbtx-AChBP interface (PDB: 1YI5) and αBgtx-nAChR interface (PDB: 9GU0), respectively. Middle: Pairwise sequence alignment of αCbtx (P01391) and αBgtx (P60615) with residue-level similarity indicated by color (green = identical, yellow = conservative substitution, coral = radical substitution, grey = indel). Bottom: Quantitative comparison showing that poly-specific V_HHs preferentially target conserved residues, while avoiding radical changes. Epitope frequency represents the fraction of V_HHs for which a given position was predicted within 4 Å of the V_HH structure in AF3-predicted complexes (true-positive predictions only, ipTM > 0.8). These results indicate that poly-specific V_HHs preferentially target conserved epitopes overlapping with receptor-binding interfaces, consistent with an association between sequence conservation and poly-specificity.

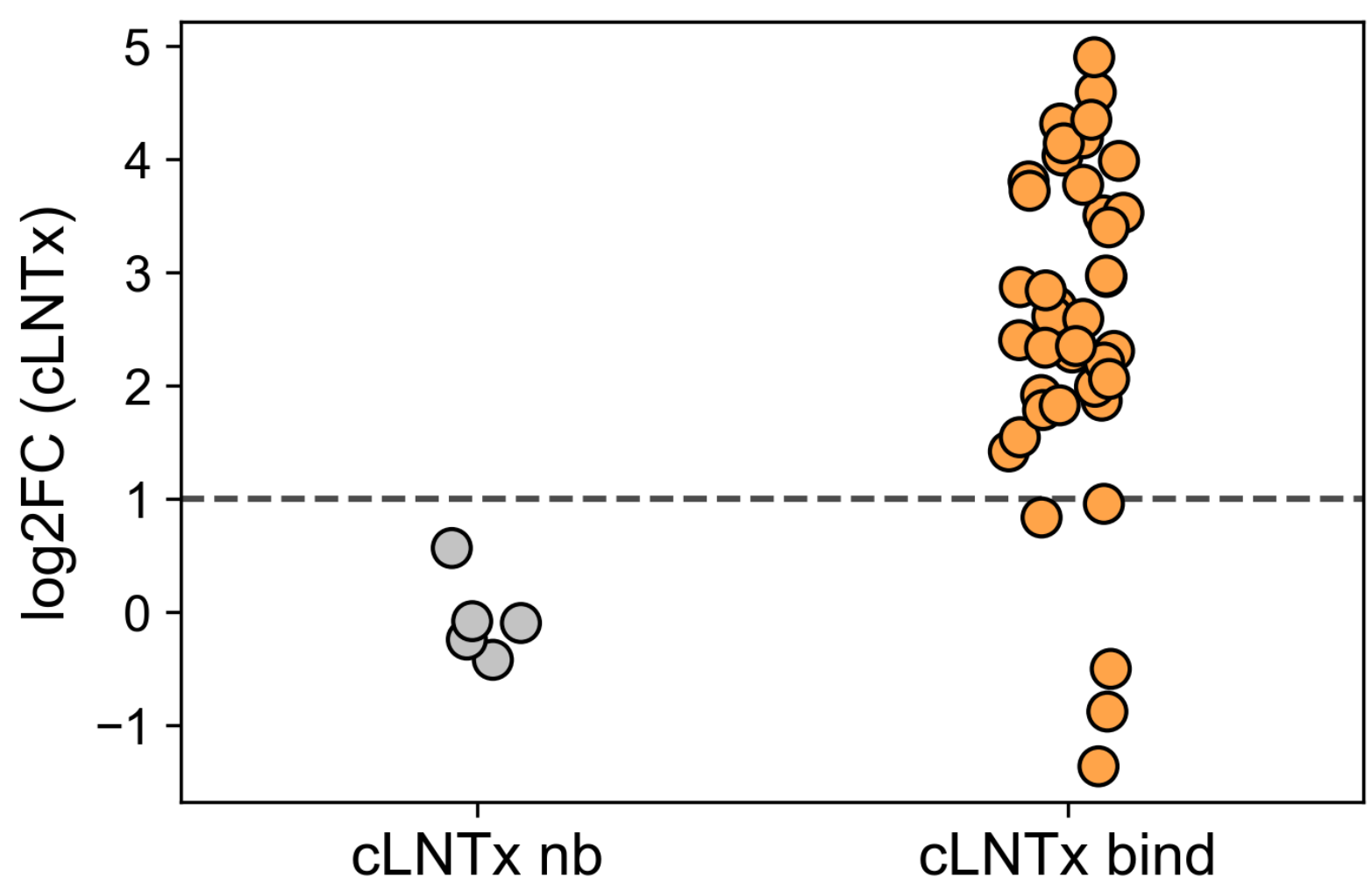

Fig. S7. **Split-luciferase cLNTx binding cutoff selection.** Split-luciferase binding signals (log_2FC) for V_HH-LargeBiT lysates measured with biotinylated cLNTx and streptavidin-SmBiT, normalized to background (V_HH-LargeBiT + substrate only). Points are colored by yeast-display label (cLNTx non-binding, grey; cLNTx binding, orange). The dashed line marks the threshold used in this study ($log_2FC = 1$), which separates the non-binding distribution from the majority of binders.

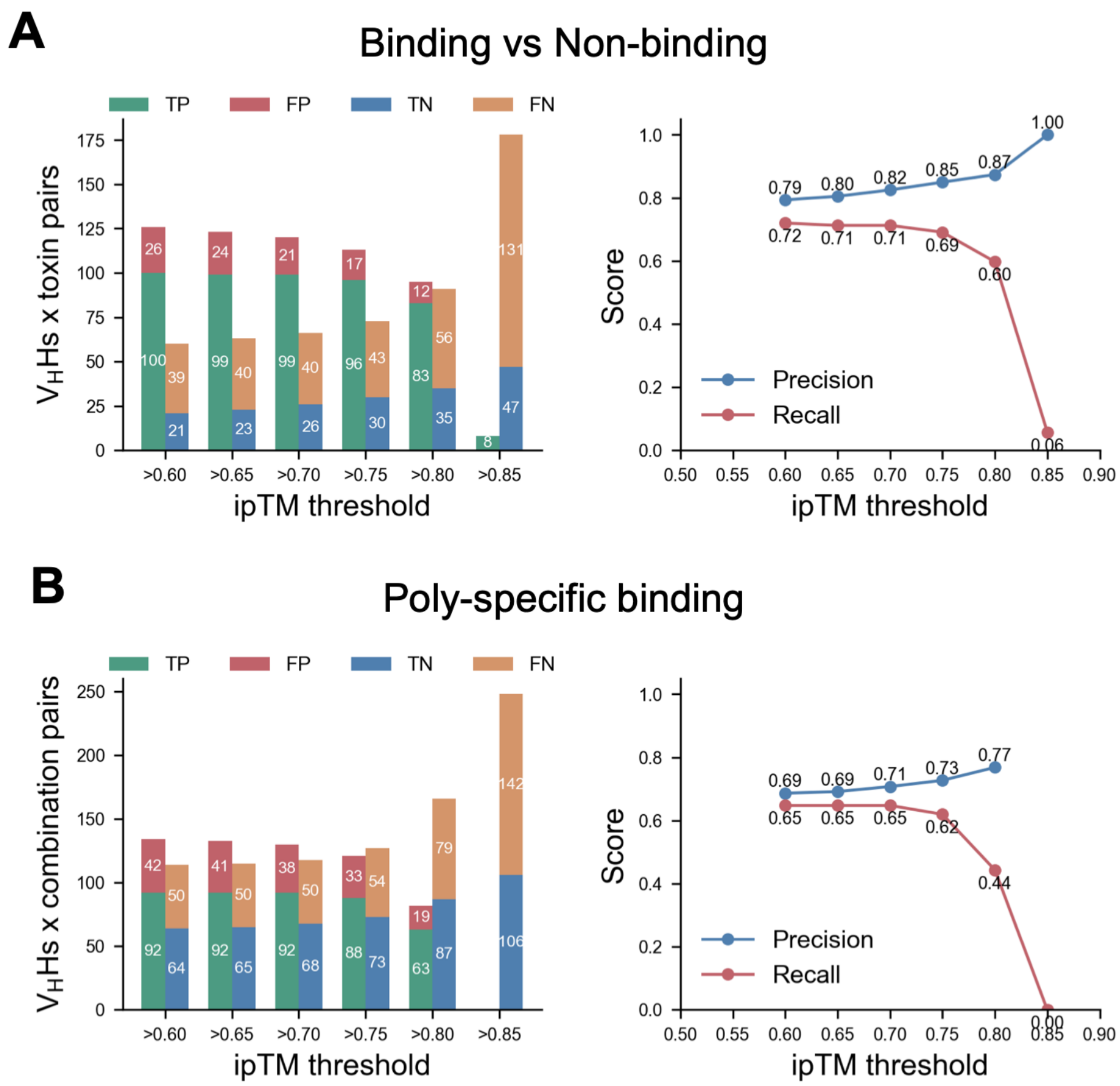

Fig. S8. ipTM threshold sensitivity analysis for toxin-binding predictions. To assess the robustness of results to the choice of ipTM cutoff, thresholds from 0.60 to 0.85 were applied to split-luciferase-validated binding (a) and poly-specific binding (b) data. For each threshold, TP/FP/TN/FN counts and corresponding precision and recall are shown. The threshold of 0.80 used throughout this study achieves high precision (0.87 for binding, 0.77 for poly-specific binding) at the cost of reduced recall, reflecting a conservative approach that prioritises minimising false positives. Performance is relatively stable across thresholds from 0.60 to 0.80, with a sharp drop in recall above 0.85. For poly-specific binding prediction specifically, a threshold of 0.75 would recover a greater fraction of true binders with only modest reduction in precision.

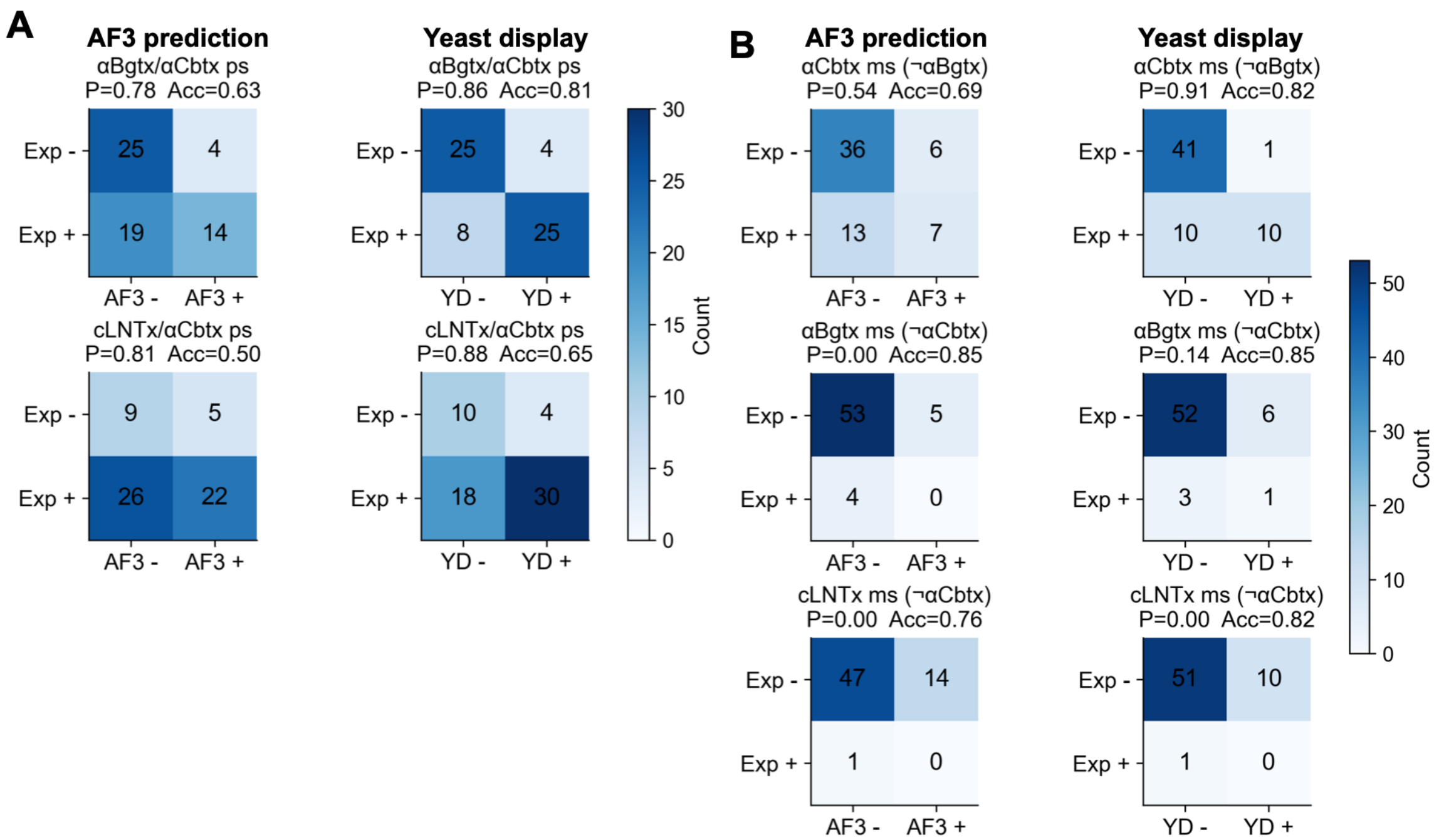

Fig. S9. Comparison of AF3 and yeast display (YD) for predicting poly- and mono-specific binding. Confusion matrices benchmark AF3 predictions (ipTM > 0.8; AF+/AF3−) and yeast display (YD) selection calls (YD+/YD−) against experimental split-luciferase binding (Exp+/Exp−; $\log_2$FC > 1). a) Poly-specific (ps) classification for αBgtx/αCbtx and cLNTx/αCbtx V_HHs. b) Mono-specific (ms) classification for αCbtx, αBgtx, and cLNTx V_HHs (¬ indicates the target that should not be bound). Precision (P) and accuracy (Acc) are shown for each panel; numbers in cells indicate counts.

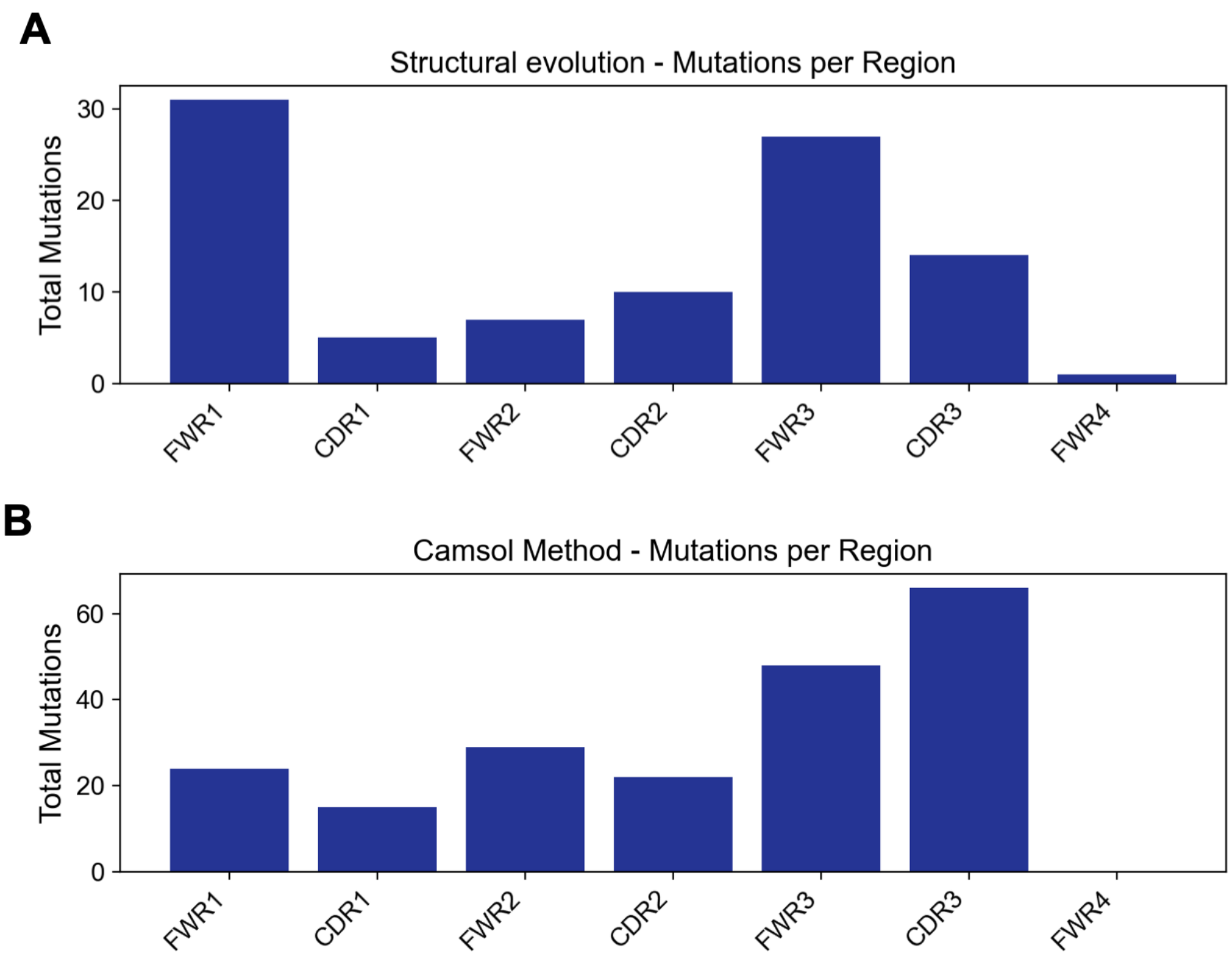

Fig. S10. Mutational distribution across V_HH regions. a) Total number of amino-acid substitutions observed in each V_HH region (FWR1–4 and CDR1–3) for the structural evolution design set. b) Same analysis for the CamSol design set. Bars report the summed mutation counts per region across all variants, highlighting distinct regional mutational biases between methods (structural evolution concentrates mutations primarily in framework regions, whereas CamSol introduces broader changes with strong enrichment in CDR3 and FWR3.

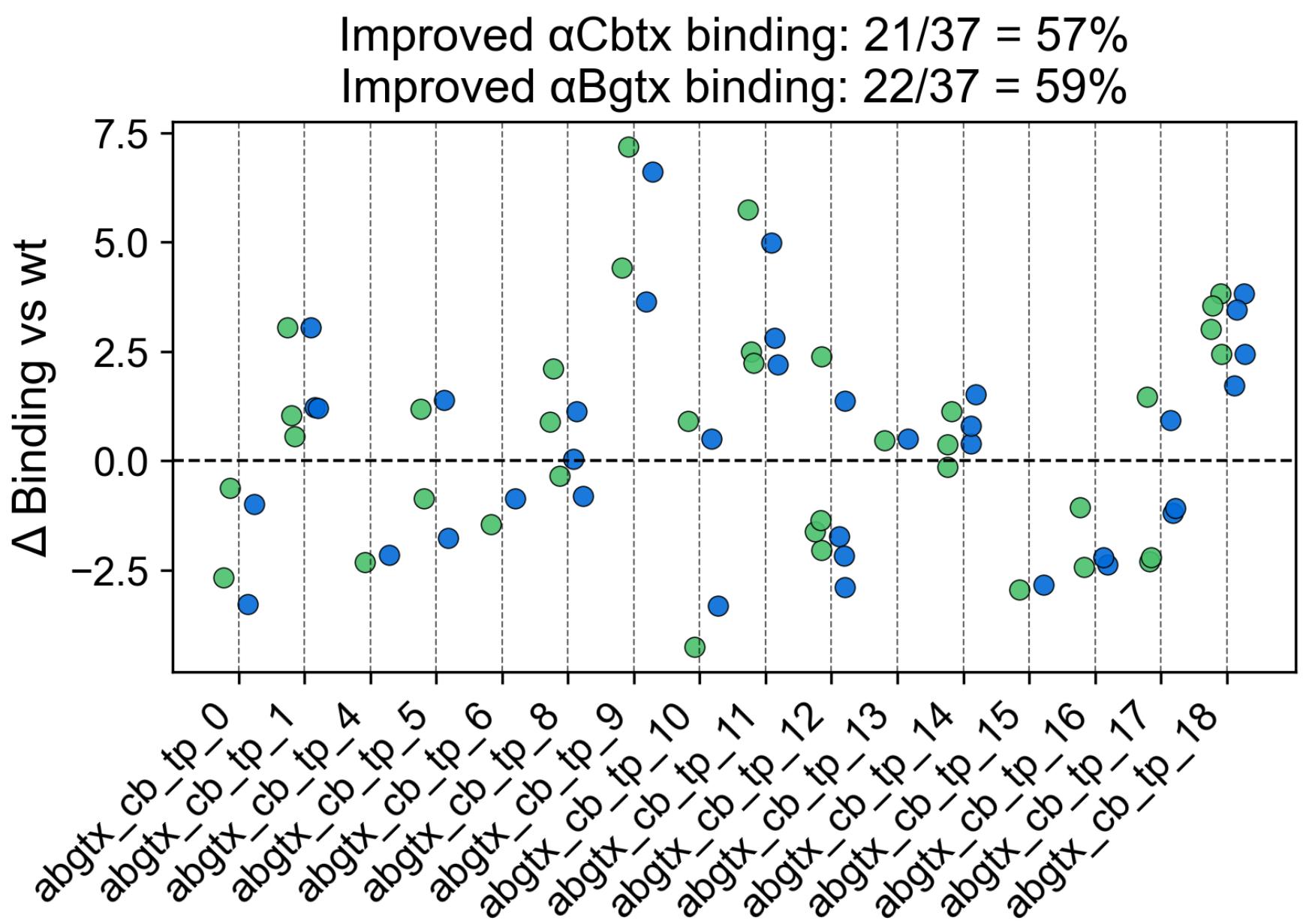

Fig. S11. CamSol variants αCbtx and αBgtx binding data. CamSol variants were also tested for binding using the Split-Luciferase binding assay. Shown is the Δ binding of variants relative to the WT. Most variants display comparable or even improved binding signals compared to the WT signal.

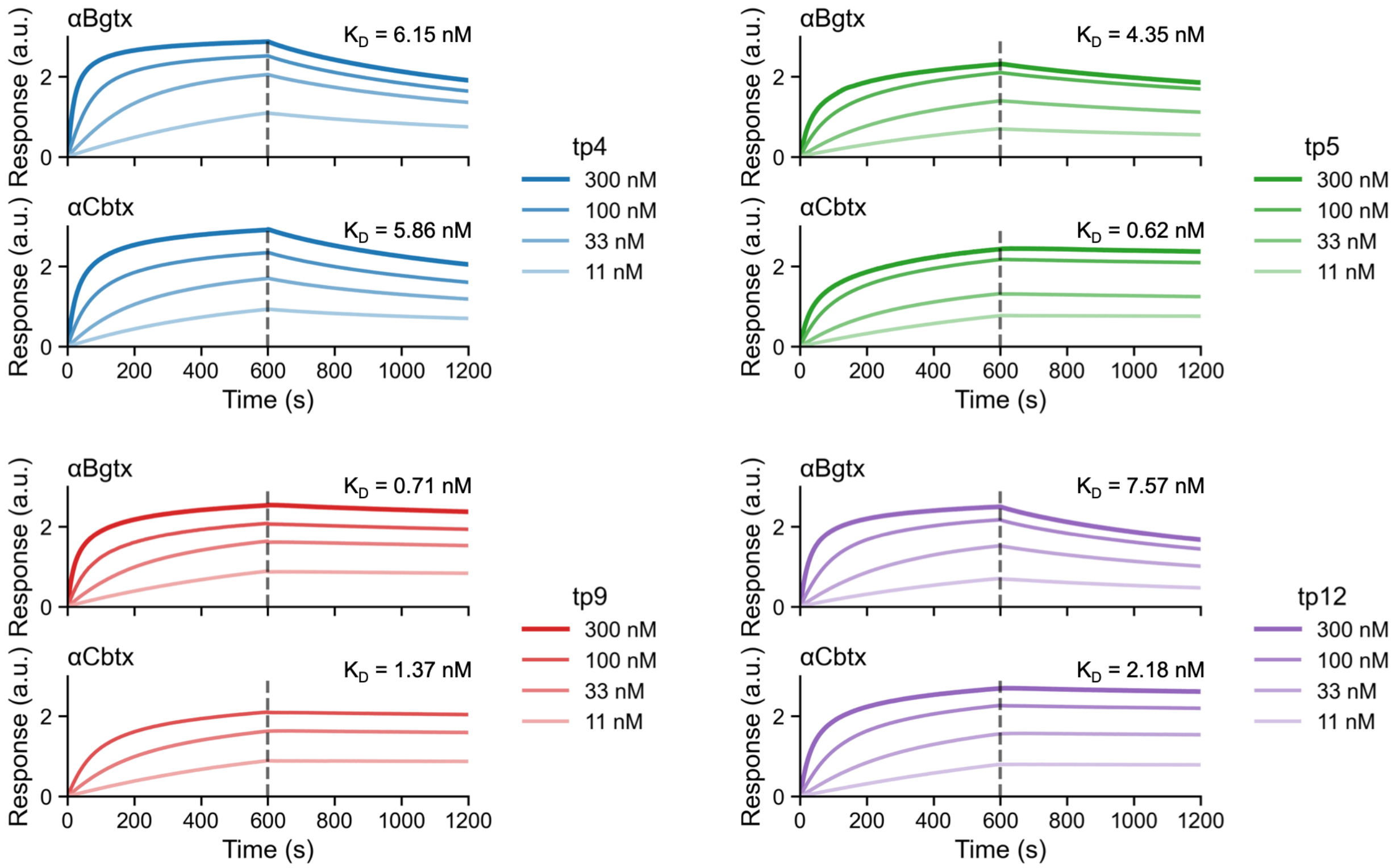

Fig. S12. BLI wild-type titrations. Association and dissociation profiles, along with K_d, are shown for V_HH tp4, tp5, tp9 and tp12 for αCBTx and αBgtx. All were tested at 4 different concentrations (300 nM, 100 nM, 33 nM and 11 nM**).**

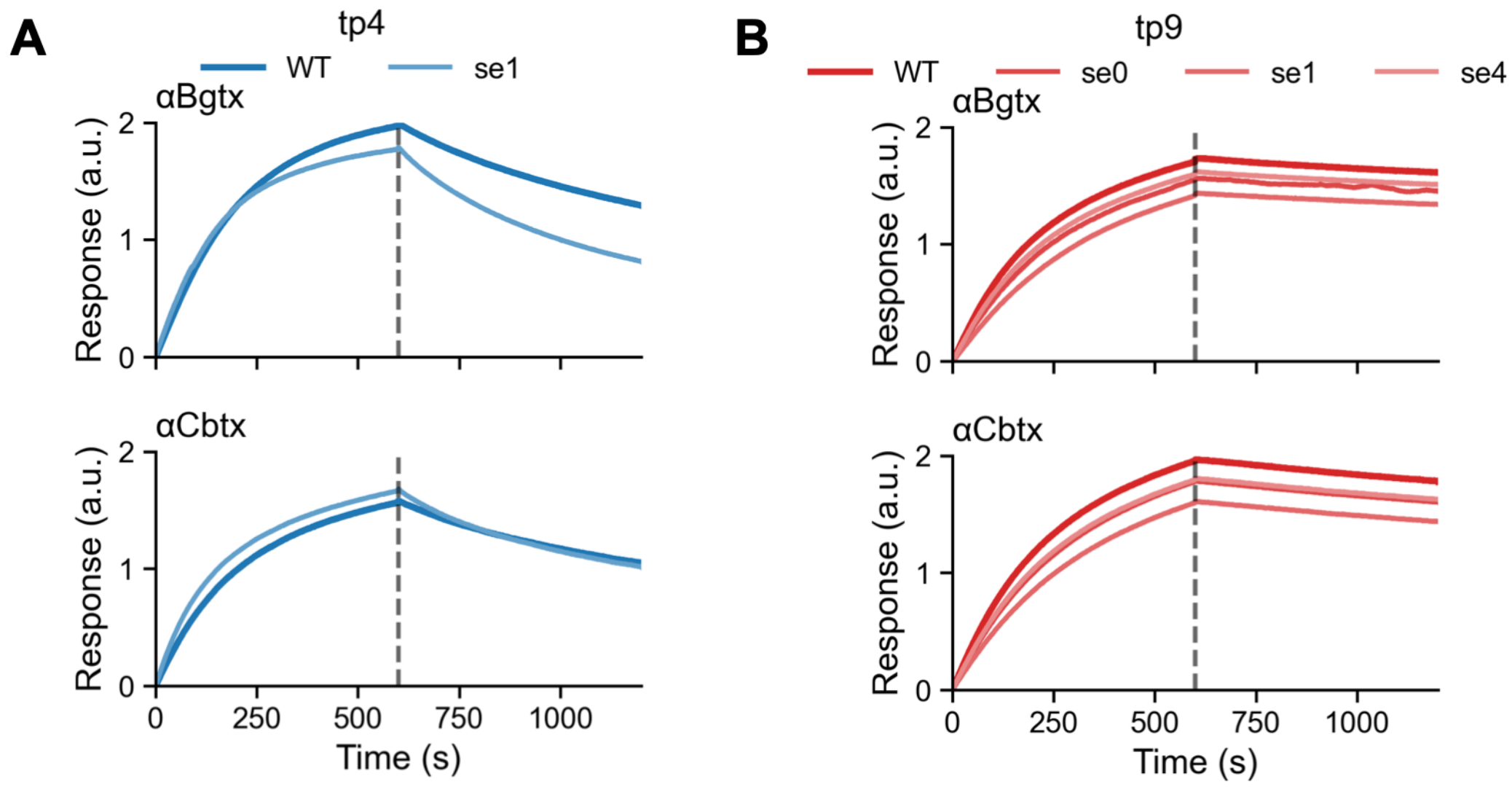

Sample ID	Target	K_D
tp4	αBgtx	6.15 nM
tp4_se1	αBgtx	9.22 nM
tp4	αCbtx	5.86 nM
tp4_se1	αCbtx	5.48 nM

Sample ID	Target	K_D (M)
tp9	αBgtx	0.71 nM
tp9_se0	αBgtx	0.98 nM
tp9_se1	αBgtx	1.27 nM
tp9_se4	αBgtx	0.78 nM
tp9	αCbtx	1.37 nM
tp9_se0	αCbtx	1.90 nM
tp9_se1	αCbtx	2.49 nM
tp9_se4	αCbtx	1.69 nM

Fig. S13. Additional BLI experiments: a) Association and dissociation profiles, along with K_d, are shown for V_HH tp4 and one tested variant (tp4_se1). This variant shows comparable affinity for αCbtx, but slightly decreased affinity for αBgtx. b) Affinity data are shown for tp9 and three tested variants. All variants display very similar, though slightly decreased, affinities for both αCbtx and αBgtx.

Data S1. structures.tar.xz

AlphaFold3 predictions output files for all predicted complexes can be found on Zenodo: https://doi.org/10.5281/zenodo.18290348

Data S2. combined_data.xlsx

Contains four sheets: (1) all V_HH sequences obtained from deep sequencing, annotated with the corresponding yeast display population; (2) all V_HH s for which AlphaFold 3 complex predictions were generated with one or more toxins. For each V_HH-toxin pair, the table reports ipTM and pTM scores from the top-ranked model; entries are NA where a given V_HH was not predicted against that toxin; (3) all V_HH s tested in the split-luciferase assay, including ipTM values for each target (αCbtx, αBgtx, cLNTx) and both raw and processed expression and binding measurements; (4) split-luciferase binding and expression data for CamSol- and structural-evolution designed V_HH variants, together with their corresponding wild-type sequences.
